# A Chromosomal Memory Triggered by *Xist* Regulates Histone Methylation in X Inactivation

**DOI:** 10.1371/journal.pbio.0020171

**Published:** 2004-07-13

**Authors:** Alexander Kohlmaier, Fabio Savarese, Monika Lachner, Joost Martens, Thomas Jenuwein, Anton Wutz

**Affiliations:** **1**Research Institute of Molecular PathologyViennaAustria

## Abstract

We have elucidated the kinetics of histone methylation during X inactivation using an inducible *Xist* expression system in mouse embryonic stem (ES) cells. Previous reports showed that the ability of *Xist* to trigger silencing is restricted to an early window in ES cell differentiation. Here we show that this window is also important for establishing methylation patterns on the potential inactive X chromosome. By immunofluorescence and chromatin immunoprecipitation experiments we show that histone H3 lysine 27 trimethylation (H3K27m3) and H4 lysine 20 monomethylation (H4K20m1) are associated with *Xist* expression in undifferentiated ES cells and mark the initiation of X inactivation. Both marks depend on *Xist* RNA localisation but are independent of silencing. Induction of *Xist* expression after the initiation window leads to a markedly reduced ability to induce H3K27m3, whereas expression before the restrictive time point allows efficient H3K27m3 establishment. Our data show that *Xist* expression early in ES cell differentiation establishes a chromosomal memory, which is maintained in the absence of silencing. One consequence of this memory is the ability to introduce H3K27m3 efficiently after the restrictive time point on the chromosome that has expressed *Xist* early. Our results suggest that this silencing-independent chromosomal memory has important implications for the maintenance of X inactivation, where previously self-perpetuating heterochromatin structures were viewed as the principal form of memory.

## Abstract 2

In female mammals, one X chromosome is inactivated. Anton Wutz and colleagues find evidence for a very early event that 'marks' the chromosome for future stable inactivation

## Introduction

In mammals, dosage differences of X-linked genes between XX female and XY male cells are adjusted by transcriptional inactivation of one of the two female X chromosomes. X inactivation is a multistep process, in which the cell counts the number of X chromosomes, chooses one to be active, and silences all others. Initiation of silencing is triggered by accumulation of the 17-kb noncoding *Xist* RNA (Borsani et al. 1991; Brockdorff et al. 1991; Brown et al. 1991). Remarkably, *Xist* RNA attaches to chromatin and spreads from its site of transcription in *cis* over the entire inactive X chromosome (Xi), mediating transcriptional repression. *Xist* is essential for initiation of silencing, but not for the maintenance of transcriptional repression on the Xi at later stages of cellular differentiation (Penny et al. 1996; Marahrens et al. 1998; Csankovszki et al. 2001). Presently, the molecular nature of the silencing mechanism is not known. Previous studies have shown that X-chromosome inactivation involves the progressive recruitment of a variety of different factors and posttranslational modifications of lysine residues in the amino termini of histones (reviewed in Brockdorff 2002). The current view is that *Xist* expression initiates the formation of heterochromatin on the Xi, which can be perpetuated by redundant silencing mechanisms at later stages. Consistent with this view, it has been shown that the Xi in mouse embryonic fibroblasts is kept inactive in the absence of *Xist* by redundant mechanisms, including DNA methylation and histone H4 hypoacetylation (Csankovszki et al. 2001).

The Polycomb group proteins Ezh2 and Eed localise to the Xi in embryonic and extraembryonic tissues early in mouse development (Wang et al. 2001; Mak et al. 2002; Plath et al. 2003; Silva et al. 2003). The human EZH2/EED and its homologous E(z)/ESC complex in Drosophila melanogaster show intrinsic histone H3 lysine 9 (H3-K9) and lysine 27 (H3-K27) methyltransferase activity (Cao et al. 2002; Czermin et al. 2002; Kuzmichev et al. 2002; Muller et al. 2002). Interestingly, H3-K27 methylation is one of the earliest chromosomal modifications on the Xi (Plath et al. 2003), and the requirement of Eed for histone methylation on the Xi has been demonstrated (Silva et al. 2003). However, analysis of Eed mutant embryos suggests that Eed is not required for initiation of silencing in trophoblast cells but is required for the maintenance of the Xi at later stages (Wang et al. 2001). Although data are consistent with the interpretation that *Xist* RNA recruits the Ezh2/Eed complex, thereby introducing histone H3 methylation, the significance of H3-K27 methylation for chromosomal inactivation is unclear. In flies, methylation on H3-K27 facilitates the binding of Polycomb to amino-terminal fragments of histone H3 (Cao et al. 2002; Min et al. 2003). Polycomb recruitment to the Xi has not been observed, and current models suggest that H3-K27 methylation in X-chromosome inactivation is indepen-dent of classical Polycomb silencing (Mak et al. 2002; Silva et al. 2003).

We have previously shown that chromosomal silencing can be recapitulated in embryonic stem (ES) cells by expressing *Xist* RNA from cDNA transgenes integrated into autosomes and the X chromosome (Wutz and Jaenisch 2000), and this allowed for an uncoupling of *Xist* regulation from cellular differentiation. In this transgenic system, *Xist* expression is under the control of a tetracycline-responsive promoter, which can be induced by the addition of doxycycline to the culture medium. We showed that *Xist* RNA localisation and silencing can be separated by introducing specific mutations in *Xist* RNA (Wutz et al. 2002). Initiation of silencing depends on the repeat A sequence at the 5′ end of *Xist.* Deletion of this element results in an RNA that localises to chromatin and spreads over the chromosome, but does not trigger transcriptional repression. Initial silencing in ES cells is reversible and dependent on *Xist* expression. At a later stage in differentiation this silent state becomes irreversible and independent of *Xist,* corresponding to the maintenance phase of X inactivation. We also showed that *Xist* expression must be induced early in ES cell differentiation to cause transcriptional repression (Wutz and Jaenisch 2000). Therefore, establishment of silencing is restricted to an initiation window in ES cell differentiation, and induction of *Xist* expression at a time point later than 24 h in differentiation no longer causes silencing. We found that *Xist* RNA loses its potential to initiate transcriptional repression roughly 24 h earlier in differentiation than the point at which silencing becomes irreversible. Notably, this left a gap of approximately one cell cycle in length between the initiation and maintenance phases. How silencing is maintained during this period and how the silent state becomes irreversible remained previously unexplained. In this report we perform kinetic measurements and quantification of histone H3 lysine 27 trimethylation (H3K27m3), revealing a novel chromosomal memory that is established by *Xist* expression at an early time point in ES cell differentiation independent of transcriptional repression. Our analysis suggests that this chromosomal memory might have an important role in the transition from the initiation phase to the maintenance phase of X inactivation.

## Results

### Profiling Histone Modification States at the Initiation of X Inactivation

**Figure 1:**
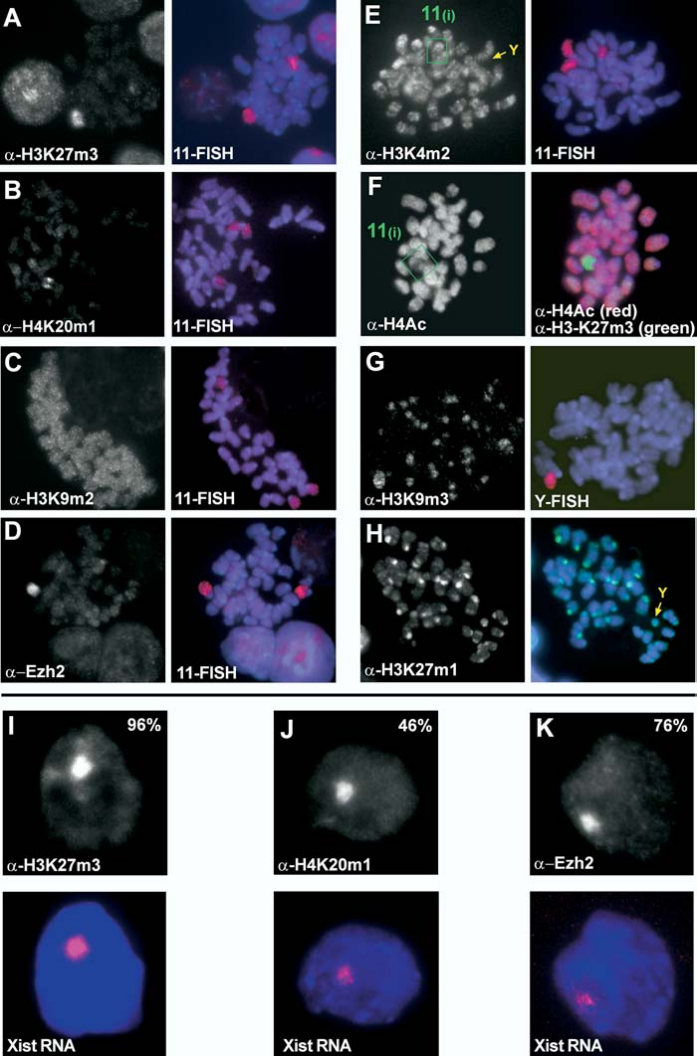
Epigenetic Imprints at the Initiation of X Inactivation (A–H) Indirect immunofluorescence and subsequent DNA FISH analysis on mitotic chromosomes prepared from undifferentiated clone 36 ES cells after 3 d of *Xist* induction. H3K27m3 (A), H4K20m1 (B), and Ezh2 (D) are enriched on the arms of Chromosome 11 upon ectopic *Xist* expression. H3K9m2 (C) is not enhanced upon *Xist* expression. H3K4m2 (E) is reduced on Chromosome 11 upon *Xist* induction (green box) and absent from pericentric heterochromatin and the Y chromosome (orange arrow). (F) Histone H4 multiple-lysine acetylation is partially reduced (green box, left panel). Hypoacetylation (red) is restricted to chromosomal regions which show high levels of H3-K27 trimethylation (green, right panel). H3K9m3 (G) and H3K27m1 (H) are enriched at constitutive heterochromatin of pericentric regions and the Y (orange arrows). (I–K) Indirect immunofluorescence (upper panels) and subsequent *Xist* RNA FISH (red, *Xist* RNA; blue, DAPI) analysis of H3K27m3 (I), H4K20m1 (J), and Ezh2 (K) in interphase nuclei of undifferentiated clone 36 ES cells expressing *Xist* for 3 d.

We have previously reported that the initial steps of chromosomal silencing in mammalian X inactivation can be recapitulated in transgenic undifferentiated male ES cells (Wutz and Jaenisch 2000). Such ES cells are useful for studying the function of *Xist* RNA in the initiation of chromosomal silencing and for analysing the kinetics and relevance of chromosomal modifications. We aimed to delineate a pattern of histone methylation states that define the initial decision for facultative heterochromatin. To achieve this we performed immunofluorescence staining against the various modification states on histone H3 and H4 lysine residues in clone 36 ES cells, in which *Xist* expression can be induced from a transgene integrated on Chromosome 11 by addition of doxycycline to the culture medium (Wutz and Jaenisch 2000). We used highly specific antisera for a defined methylation state (mono-, di-, or tri-) at a particular lysine residue in the amino terminus of histone H3 and H4 (Peters et al. 2003; Perez-Burgos et al. 2004). Some cross reactivity of the H3K27m2 antiserum with H3K27m1 and H3K27m3, of the H3K4m3 antiserum with H3K4m2, and of the H4K20m2 antiserum with H4K20m1 and H4K20m3 was detected on peptide blots (Figure S1), but does not affect the conclusions drawn in this study. Our cytological experiments show a focal signal for H3K27m3 in the interphase nuclei of clone 36 ES cells upon *Xist* expression, which colocalises with Chromosome 11 in metaphase spreads and *Xist* RNA in interphase nuclei (Figure 1). In cells grown in the absence of doxycycline, a diffuse nuclear signal was observed. H3-K27 mono- and dimethylation were equally present on the inactivated chromosome and other autosomes (Table 1). Notably, we did not observe any specific enrichment for the H3K9m1, H3K9m2, or H3K9m3 signal on Chromosome 11 upon *Xist* induction (Figures 1C, 1G, and S2). H3K9m3 and H3K27m1 colocalised strictly with constitutive heterochromatin at pericentric regions and the Y chromosome (Figure 1G and 1H). H3K4m2 and H3K4m3 gave banded signals on chromosome arms that were reduced but not entirely erased on the transgenic chromosome, when *Xist* expression was induced (Figures 1E and S2J). The heterochromatic Y chromosome completely lacked both H3K4m2 and H3K4m3 in the same metaphase spread. Thus, we conclude that the reduction of H3K4m2 and H3K4m2 on the *Xist*-expressing chromosome is consistent with a state of transcriptional repression (Santos-Rosa et al. 2002) and with earlier reports that implicate H3-K4 hypomethylation early in X inactivation (Heard et al. 2001; O'Neill et al. 2003). H3K4m1 was equally present on the *Xist*-expressing chromosome and other autosomes. Using antisera specific for methylation states of H4K20, we observed that H4K20m1 decorated Chromosome 11 upon *Xist* induction in undifferentiated clone 36 ES cells (in 46% of interphase nuclei; Figure 1B). H4K20m2 and H4K20m3 were not enriched on the *Xist*-expressing chromosome (Figure S2H and S2I; G. Schotta and M. Lachner, unpublished data). We also investigated the acetylation state of histone H4 in these cells using a sheep polyclonal antiserum that preferentially recognises multiply acetylated H4 (Morrison and Jeppesen 2002). Using this antiserum, we detected partial hypoacetylation of Chromosome 11 in metaphase spreads of clone 36 ES cells that were induced to express *Xist* (Figures 1F, S2K, and S2L). This observation is different from the global chromosome-wide hypoacetylation of H4 that was reported on the Xi later in differentiation (Keohane et al. 1996) and might reflect the absence of active promoters. We also detected a degree of hypoacetylation when a silencing-defective *Xist* RNA was expressed (Figure S3), making it likely to be the consequence of cross talk with H4-K20 methylation, which is mutually exclusive at least with H4-K16 acetylation (Nishioka et al. 2002). In conclusion, H3K27m3, H4K20m1, reduction of H3K4m2 and H3K4m3, and reduced multiple-lysine acetylation of histone H4 correlate with the inactive state of the chromosome in undifferentiated ES cells (Table 1).

**Table 1:**
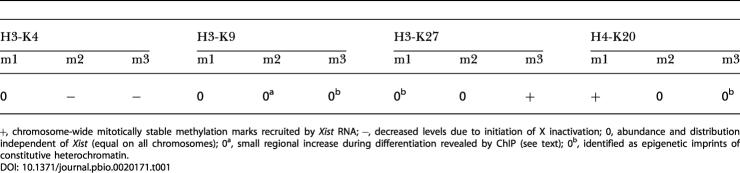
Histone Lysine Methylation States as Epigenetic Imprints during X Inactivation

+, chromosome-wide mitotically stable methylation marks recruited by *Xist* RNA; −, decreased levels due to initiation of X inactivation; 0, abundance and distribution independent of *Xist* (equal on all chromosomes); 0^a^, small regional increase during differentiation revealed by ChIP (see text); 0^b^, identified as epigenetic imprints of constitutive heterochromatin

**Figure 2:**
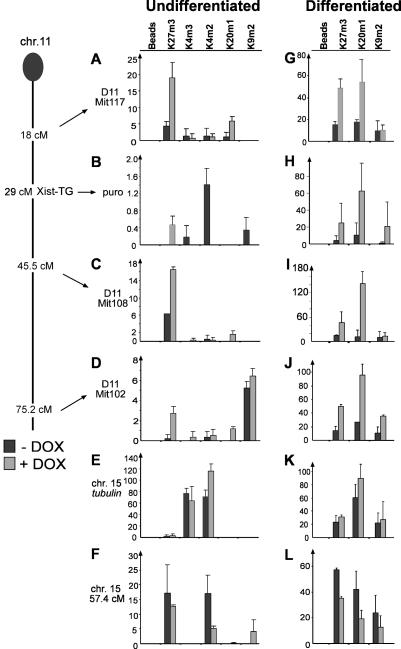
ChIP Mapping of H3K27m3, H4K20m1, H3K9m2, H3K4m3, and H3K4m2 on the *Xist*-Expressing Chromosome 11 during Differentiation of Clone 36 ES Cells A genetic map of Chromosome 11 indicating the loci analysed is given on the left (*Xist*-TG, approximate integration site of *Xist* transgene; *puro, PGKpuromycin* marker). (A to F) Chromatin was prepared from undifferentiated clone 36 ES cells grown for 3 d in the presence (light bars) or absence (dark bars) of doxycycline. H3K27m3 and H4K20m1 were enriched at three intergenic microsatellite sequences at 18.0 (A), 45.5 (C), and 75.2 (D) cM. (B) H3K27m3 was established over the coding sequence of *PGKpuromycin* in doxycycline-induced cells, which was accompanied by a loss of H3K4m2 and H3K4m3. (E) Tubulin control. (F) Control microsatellite located on Chromosome 15. (G–L) Analysis of H3K27m3, H4K20m1, and H3K9m2 in clone 36 ES cells differentiated for 9 d with (light bars) or without (dark bars) doxycycline. Histone methylation marks were monitored. Experiments were performed in duplicate, and the standard error is indicated in the graphs.

Further confirmation of the cytological findings comes from chromatin immunoprecipitation (ChIP) experiments using antibodies specific for H3K27m3, H4K20m1, H3K4m3, H3K4m2, and H3K9m2 in both undifferentiated and differentiated clone 36 ES cells in the presence or absence of doxycycline (Figure 2). We observed enhanced H3K27m3 and H4K20m1 in the cells expressing *Xist* regardless of the differentiation state on three microsatellite sequences on Chromosome 11 (Figure 2). A control microsatellite on Chromosome 15 did not show this effect (Figure 2F and 2L). Upon *Xist* expression, we also observed H3K27m3 on the puromycin marker gene cointegrated with the *Xist* transgene on Chromosome 11, compared to nearly undetectable levels in the uninduced sample (Figure 2B). This increase in H3K27m3 was paralleled by a marked decrease in H3K4m2 and H3K4m3, but no increase in H4K20m1 could be observed at this locus in undifferentiated ES cells. Upon differentiation, an increase in the H4K20m1 signal was observed when *Xist* was expressed on all sequences on Chromosome 11. A control tubulin gene located on Chromosome 15 showed no significant change upon *Xist* induction (Figure 2E and 2K). These data show that H3K27m3 and H4K20m1 are elevated by *Xist* RNA expression on the transgenic chromosome, in agreement with our cytological analysis. However, regional differences are revealed by the higher resolution of the ChIP experiment, showing that the two modifications do not display a completely overlapping distribution on the chromosome. Differentiation of the ES cells resulted in increased H4K20m1 signals dependent on *Xist* expression. H3K9m2 was also elevated on two loci on Chromosome 11.

### H3K27m3 and H4K20m1 Are Triggered by *Xist* RNA Localisation and Are Independent of Silencing

In agreement with earlier studies (Plath et al. 2003; Silva et al. 2003), our results indicate that chromosome-wide histone H3K27m3 is efficiently triggered in undifferentiated ES cells and therefore is an early mark of X inactivation. We measured the kinetics of H3K27m3 following induction of *Xist* RNA expression in undifferentiated clone 36 ES cells (Figure S3A). At 6, 12, and 24 h after induction 0%, 12%, and 37% of the cells, respectively, showed a signal, and by 48 h a maximum of 70% was reached. Furthermore, the recruitment of Ezh2 protein to the transgenic Chromosome 11 upon *Xist* expression (see Figure 1D and 1K) is consistent with the idea that the Ezh2/Eed complex contains the enzymatic activity causing H3K27m3 in X inactivation (Mak et al. 2002; Plath et al. 2003; Silva et al. 2003).

**Figure 3:**
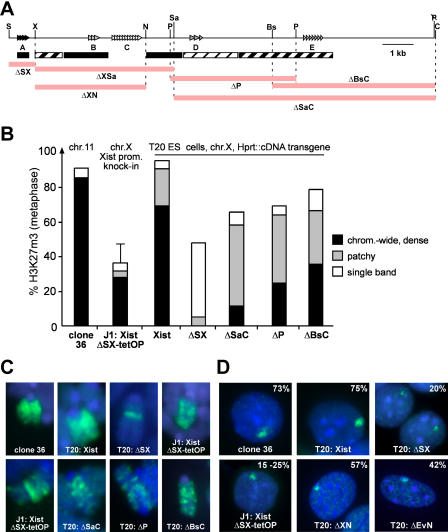
Sequences of *Xist* RNA Required for H3K27m3 Establishment (A) Schematic representation of the *Xist* cDNA (top) indicating repeats A to E, restriction sites, and the locations of deletions (coloured bars) relative to the location of sequences required for localisation (black and hatched boxes; Wutz et al. 2002). (B) Analysis of H3K27m3 on metaphase chromosome spreads from undifferentiated ES cells after 3 d of *Xist* induction (see text). The staining patterns (*n* > 100) were scored as chromosome-wide dense methylation (black), reduced methylation (grey), and a single band (open). (C) Pattern of H3K27m3 triggered by different *Xist* mutants on metaphase chromosomes after 3 d of induction. Enlarged view of Chromosome 11 (clone 36) or the X chromosome (T20 lines, J1 knock-in line). (D) Focal H3K27m3 staining in interphase nuclei (percentage given; *n* > 100) of undifferentiated ES cells expressing *Xist* constructs.

To identify the *Xist* sequences that are required for the binding of the Ezh2/Eed complex and to trigger H3K27m3, we examined a panel of *Xist* RNA mutations (Figure 3A). In an earlier study we inserted *Xist* cDNA transgenes containing defined deletions into the *Hprt* gene locus on the single X chromosome in male mouse T20 ES cells and measured their ability to cause silencing (Wutz et al. 2002). We used deletions spanning the entire RNA that eliminate relatively large parts of *Xist* to analyse H3K27m3 by immunofluorescence in ES cells after induction of transgenic *Xist* expression (Figure 3). H3K27m3 staining was observed for all *Xist* mutations tested, with the exception of the ΔXSa deletion, where sequences required for localisation are deleted. The resulting XistΔXSa RNA did not localise well to chromatin and showed consequently greatly diminished potential to silence (Figure S4). We interpret the absence of detectable H3K27m3 in this case as a consequence of the failure of the RNA to localise. All other mutants analysed, including that containing a ΔXN deletion spanning a similar region, gave rise to RNA that localised well to chromatin and caused H3K27m3. A mutant with a deletion of repeat A (T20:ΔSX ES cells; Figure 3), which localises to chromatin but does not cause silencing, was able to induce H3K27m3, suggesting that methylation can be established independent of silencing, a finding consistent with the results of an earlier study (Plath et al. 2003). The expression of the silencing-deficient *Xist* RNA led to a significantly lower percentage of cells with H3K27m3 foci in interphase nuclei (3- to 4-fold reduction compared to wild-type *Xist* RNA; Figure 3D). Moreover, on metaphase chromosomes methylation appeared mostly as a single band (only 5% showed a wild-type pattern; Figure 3C). Since the transgene is integrated in the *Hprt* locus on the X chromosome and the endogenous *Xist* gene is still present in this cell line, the possibility exists that the transgenic RNA might have stabilised the endogenous *Xist* RNA or vice versa to effect H3K27m3. To address this point we made use of another cell line in which repeat A was deleted from the endogenous *Xist* gene and an inducible promoter was inserted by homologous recombination (J1:XistΔSX-tetOP; Wutz et al. 2002). Induction of *Xist* RNA expression caused H3K27m3 on the single X chromosome in these cells, confirming that H3K27m3 can be established by *Xist* expression in complete absence of repeat A sequences. However, in undifferentiated ES cells, expression of the silencing-deficient *Xist* RNA led consistently to lower numbers of cells (30%–35%) showing H3K27m3 staining compared to the wild-type *Xist* RNA (80%; Figure 3B and 3C). Mono- and dimethylation of H3-K27 were not visibly elevated in J1:XistΔSX-tetOP cells at the expense of the H3K27m3 signal (data not shown), suggesting that recruitment of the Ezh2/Eed complex was impaired in the absence of repeat A, and ruling out the possibility that repeat A would change the specificity of the complex to induce trimethylation activity. Consistent with this interpretation, Ezh2 was observed in only 9% of the J1:XistΔSX-tetOP ES cells compared to 76% of the clone 36 ES cells (see Figures 1K and S3D). We note that the lower methylation potential of *Xist* RNA lacking repeat A sequences was only observed in undifferentiated ES cells. When the cells were differentiated, methylation levels were elevated (see Figure S3C). We further determined the role of H4K20m1 in silencing. We detected H4K20m1 upon induction of *Xist* expression in 14% of the interphase nuclei in undifferentiated J1:XistΔSX-tetOP ES cells, showing that H4K20m1 can be established in the absence of repeat A (see Figure S3D). We conclude that H3K27m3 and H4K20m1 are independent of and not sufficient for silencing.

### Efficient H3K27m3 Is Restricted to Early Stages of Differentiation

**Figure 4:**
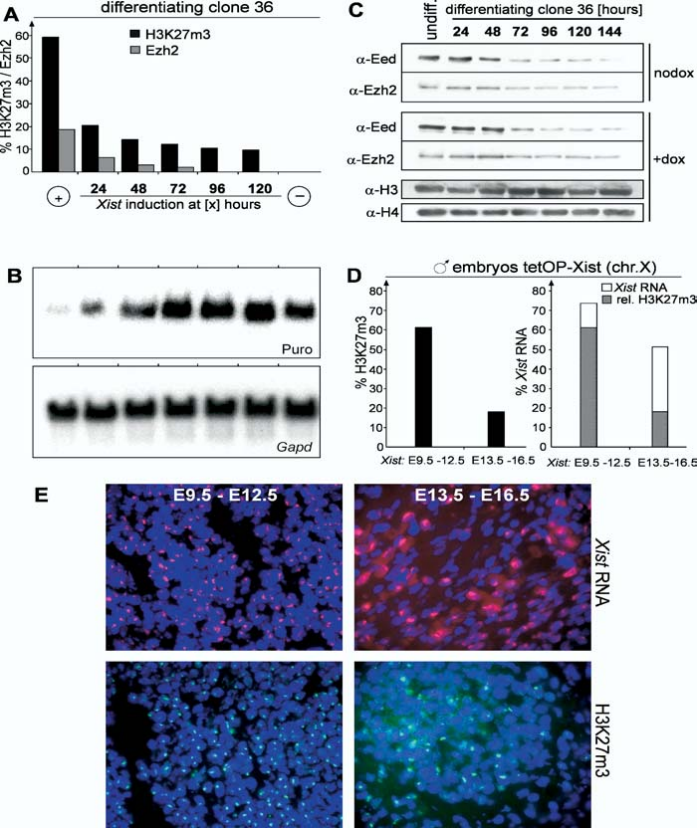
Restriction of H3K27m3 Establishment and Transcriptional Silencing in Differentiation (A) Initiation of H3K27m3 during clone 36 ES cell differentiation. *Xist* expression was induced at the beginning (+) or at various time points (24 to 120 h) after the start of differentiation, or not induced (−). The percentages of interphase cells showing H3K27m3 (black bars; *n* > 700) and Ezh2 (grey bars; *n* > 200) staining were determined at day 12 of differentiation. (B) Initiation of transcriptional silencing during differentiation was assessed by Northern blot analysis of *PGKpuromycin (puro)* and *Gapd* as a loading control in parallel cultures as described for (A). (C) Western analysis of Ezh2 and Eed protein levels during differentiation of clone 36 ES cells after induction with retinoic acid. Histones H3 and H4 were used as a loading control. (D) Establishment of H3K27m3 during embryonic development. *Xist* expression was induced from the single X chromosome of male Xist-tetOP embryos (see text) for 3 d (E9.5–12.5 and E13.5–16.5). The percentage of cells with H3K27m3 staining in interphase (left) and clusters of *Xist* RNA (right, open bars) are given (*n* > 300). Grey areas indicate the proportion of H3K27m3-positive cells to *Xist*-positive cells. (E) *Xist* RNA FISH (top) and H3K27m3 (bottom) staining of histological sections prepared from neck connective tissue of embryos described in (C).

*Xist*-mediated transcriptional silencing is restricted in ES cell differentiation in that the potential of *Xist* to initiate repression diminishes 48 h after differentiation (Wutz and Jaenisch 2000). We investigated whether the ability to establish H3K27m3 would be restricted to this initiation window in clone 36 ES cells. These cells carry a puromycin resistance gene *(puro),* which is cointegrated with the *Xist* cDNA transgene on Chromosome 11 and can be silenced by transgenic *Xist* expression. *Xist* expression was induced either from the beginning or at 24, 48, 72, 96, or 120 h after the onset of differentiation. The ability of *Xist* to initiate silencing at various time points was monitored by measuring *puro* expression, and H3K27m3 was analysed in parallel in all cultures at 12 d after differentiation (Figure 4A). When *Xist* was induced within 24 h of differentiation, H3K27m3 was observed in a large fraction of the cells. Induction of *Xist* after 24 h led to significantly lower methylation levels (10%–15% of cells; Figure 4A). The efficiency in H3K27m3 pattern establishment correlated at all time points with the potential of *Xist* to initiate silencing and Ezh2 recruitment (Figure 4B). Hence, an efficient H3K27m3 pattern was established in a time window that overlapped with the window for the initiation of *Xist*-mediated repression. We also determined the levels of Eed and Ezh2 protein during ES cell differentiation (Figure 4C). Our analysis shows that Eed levels are significantly reduced at day 3 of differentiation and Ezh2 levels diminish more gradually towards even later time points. This demonstrates that the ability of *Xist* to induce efficient H3K27m3 is restricted at a time when both Eed and Ezh2 proteins are detected in similar amounts, as in undifferentiated ES cells, suggesting that the efficiency of methylation is not a function of the protein levels.

To confirm this finding, we assayed the effect of induction of *Xist* expression on H3K27m3 in embryonic fibroblasts. Fibroblasts were isolated from male, day 13.5 embryos carrying an insertion of the doxycycline-inducible promoter in the endogenous *Xist* locus (Xist-tetOP allele) and a homozygous insertion of the tetracycline-responsive transactivator in the ROSA26 locus (ROSA26-nlsrtTA allele; Wutz et al. 2002; F. Savarese, unpublished data). In these fibroblasts, expression of the endogenous *Xist* RNA from the single male X chromosome could be induced in 80% of the cells by addition of doxycycline (data not shown). In uninduced cultures and control male fibroblasts no H3K27m3 foci were detected by immunofluorescence in interphase nuclei. However, upon *Xist* induction 5% (after 48 h of *Xist* induction) or 15% (after 72 h) of the cells showed focal H3K27m3 staining (H4K20m1 was established, as well; see Figure S3G). In control female fibroblasts H3K27m3 staining was detected in 85% of the cells. This shows that *Xist* induction in embryonic fibroblasts leads to H3-K27 methylation in a low percentage of cells. We further examined histological sections of male embryos carrying the inducible Xist-tetOP allele and the ROSA26-nlsrtTA allele. *Xist* expression was induced by feeding doxycycline in drinking water to the mothers for 3 d starting either from day 9.5 or day 13.5 of gestation. Embryos were dissected 3 d later, on day 12.5 and 16.5, respectively. In the sections, 74% (day 12.5 embryos) and 52% (day 16.5 embryos) of the cells expressed *Xist,* as determined by RNA fluorescent in situ hybridization (FISH) analysis (Figure 4D and 4E). Focal H3K27m3 staining was detected in 61% of the cells in sections of the day 12.5 embryos but in only 18% of the day 16.5 embryos (Figure 4D and 4E), demonstrating a clear reduction in the number of cells showing H3K27m3 staining in response to *Xist* expression in the later-stage embryos. In summary, our data demonstrate that *Xist* has been able to effect H3K27m3 in all cell types tested. However, the efficiency of methylation is regulated in cellular differentiation and development. Our experiments show that *Xist* is not sufficient for efficient establishment of the H3K27m3 pattern in differentiated cells.

### Reversibility of H3K27m3

**Figure 5:**
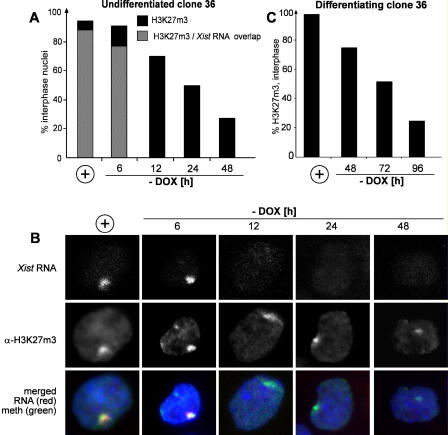
Kinetic Study of H3K27m3 Stability (A) The percentage of interphase nuclei (*n* > 100) showing H3K27m3 staining and *Xist* RNA was analysed for undifferentiated clone 36 ES cells, which expressed *Xist* for 3 d (+) or were further grown without inducer for 6, 12, 24, or 48 h. (B) Representative images of the time points analysed in (A) are shown. (C) Reversibility of H3K27m3 in differentiating clone 36 ES cells. The percentage of interphase cells showing H3K27m3 staining (*n* > 100) was determined for cells differentiated for 4 d in the presence of doxycycline (+) or further differentiated for 48, 72, or 96 h in the absence of inducer.

Once efficient H3K27m3 is established by *Xist* expression in early ES cell differentiation, it can be maintained throughout differentiation. This would be consistent with the view that lysine methylation is a permanent epigenetic mark. To test whether H3K27m3 is stably maintained in the absence of continuous *Xist* expression, we tested H3K27m3 reversibility in undifferentiated clone 36 ES cells. *Xist* expression was induced from the transgenic Chromosome 11 in these cells for 3 days, and then the cells were washed and split into medium without doxycycline to shut off *Xist* expression. H3K27m3 levels and *Xist* RNA were determined by combined immunofluorescence RNA FISH at consecutive time points at 6, 12, 24, and 48 h. High levels of H3K27m3 persisted until 24 h after *Xist* was turned off, but H3K27m3 disappeared by 48 h (Figures 5A and S3B). Our data show that the *Xist* RNA signal disappeared by 12 h after the withdrawal of doxycycline, demonstrating that H3-K27 methylation is reversible in undifferentiated ES cells and is removed after a period of approximately two cell divisions following the turning off of *Xist* expression. We also analysed the reversibility of H4K20m1 and Ezh2 in undifferentiated clone 36 ES cells. The percentage of cells showing a signal went from 46% and 70% initially to 5% and 11% at 48 h after withdrawal of doxycycline for H4K20m1 and Ezh2, respectively.

To test whether H3K27m3 would become irreversible during ES cell differentiation, we turned off *Xist* expression in clone 36 ES cells at progressively later time points up to 6 d after initiation of differentiation. The H3K27m3 pattern was analysed in all cultures at day 12 of differentiation. In cells continuously expressing *Xist* during differentiation, methylation was detected in 60% of the cells at day 12. If *Xist* expression was turned off at any time points in the course of differentiation, the percentage of cells showing H3K27m3 was reduced to less than 10%, suggesting that methylation was reversible throughout differentiation and not stabilised (data not shown). We then analysed the kinetics of loss of methylation in differentiated ES cells. Clone 36 ES cells differentiated for 4 d in the presence of doxycycline were differentiated for 24, 48, and 72 more hours in the absence of doxycycline, and H3K27m3 was measured (Figure 5C). Focal H3K27m3 staining was initially observed in 97% of interphase nuclei and was reduced to 50% and 25% at 3 and 4 d, respectively, after *Xist* had been turned off. This shows that the decay of focal H3K27m3 was slower than in undifferentiated ES cells, possibly reflecting the slower cell cycle of differentiating cells.

### Early *Xist* Expression Triggers a Chromosomal Memory Independent of Silencing

**Figure 6:**
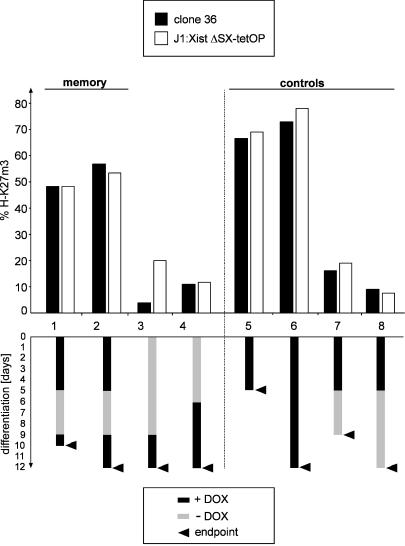
Early *Xist* Expression Imparts a Chromosomal Memory Independent of Silencing Transgenic *Xist* expression was induced from Chromosome 11 in clone 36 ES cells (black bars) or a silencing-deficient *Xist* RNA from the X in J1:XistΔSX-tetOP ES cells (open bars) at time points during differentiation (see text). The percentage of cells showing H3K27m3 staining is plotted (*n* > 250). Below, a scheme of *Xist* induction is given for all cultures, with arrows representing time of analysis.

Detection of focal H3K27m3 staining persisted throughout ES cell differentiation when *Xist* was continuously expressed. Yet the methylation mark was reversible throughout ES cell differentiation, and *Xist* RNA could only establish an efficient methylation pattern during the initiation window early in ES cell differentiation. These observations could indicate that silencing enhances histone methylation in ES cell differentiation. To address this interpretation, we analysed the H3K27m3 pattern caused by expression of a mutant *Xist* RNA lacking repeat A, which cannot initiate silencing, in differentiating J1:XistΔSX-tetOP ES cells. When these cells were differentiated in the presence of doxycycline, focal H3K27m3 staining was observed in 78% of the cells at day 12 (Figure 6). This clearly indicated that methylation was maintained in a high number of these cells. Silencing is therefore dispensable for methylation in ES cell differentiation. Notably, we observed H3K27m3 staining in a high percentage of differentiated ES cells but in a significantly reduced percentage of undifferentiated ES cells expressing a silencing-defective *Xist* RNA (see Figure 3B, 3C, and S3C). Silencing or repeat A sequences are therefore required to sustain high H3K27m3 levels specifically in undifferentiated ES cells but are dispensable upon differentiation.

To test whether continuous *Xist* expression was required for maintenance of efficient H3K27m3, we induced *Xist* expression from the transgenic Chromosome 11 in undifferentiated clone 36 cells and from the X chromosome in J1:XistΔSX-tetOP ES cells for 3 d. The cells were then differentiated for 5 d in the presence of doxycycline followed by 5 and 7 d, respectively, without the inducer. At the end of this period H3K27m3 was analysed and could be detected in less than 20% and 10% of the cells, respectively (Figure 6). Parallel cultures were differentiated for 5 d in the presence of doxycycline followed by 4 d in the absence of doxycycline, and then doxycycline was added back for 1 or 3 more days. In these cells, in which *Xist* had been induced early, H3K27m3 was restored and detectable in 50%–55% of all cells. This level is significantly higher than the level in control cultures that had been induced de novo at day 6 or day 9 of differentiation (10% of all cells). In cells that had been continuously differentiated in the presence of doxycycline, methylation was detected in 73%–78% of the nuclei. Our data show that efficient methylation at late time points in differentiation did not require continuous *Xist* expression. Efficient remethylation occurred on a chromosome that had been exposed to *Xist* in early ES cell differentiation, consistent with the idea that *Xist* triggers a chromosomal change in early differentiation that is remembered until later time points to enhance H3K27m3 reestablishment. Importantly, the silencing-deficient *Xist* mutant RNA in J1:XistΔSX-tetOP ES cells gave identical results, showing that this memory is established independent of silencing.

**Figure 7:**
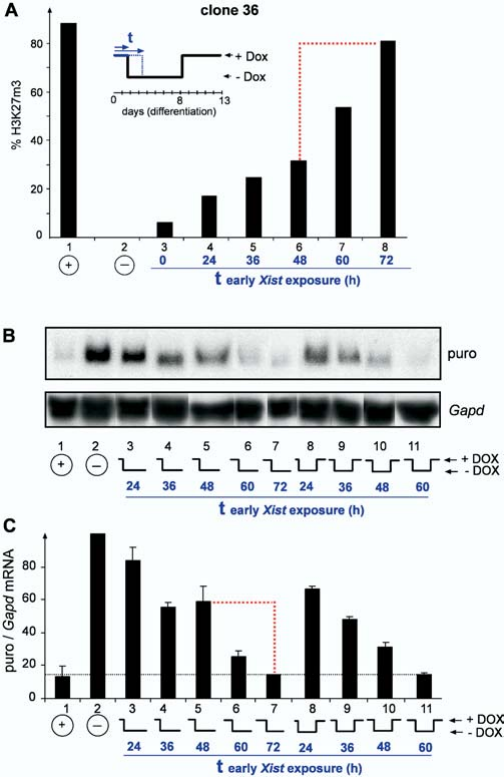
Establishment of Chromosomal Memory during ES Cell Differentiation (A) Clone 36 ES cells were differentiated for 13 d in the presence of doxycycline (lane 1) or in the absence of inducer (lane 2) and the percentage of cells with H3K27m3 staining was determined (*n* > 800). At the beginning of differentiation, parallel cultures received either no *Xist* induction (lane 3) or a pulse of doxycycline for 24 h (lane 4), 36 h (lane 5), 48 h (lane 6), 60 h (lane7), or 72 h (lane 8) followed by withdrawal of inducer and concerted late induction from day 8 to day 13. A dashed red line indicates the 24-h interval of the transition when the chromosomal memory is recruited. (B and C) Establishment of irreversible transcriptional silencing during differentiation. (B) Ectopic inactivation of Chromosome 11 caused by *Xist* induction in differentiating clone 36 ES cells was assessed by Northern blot analysis of *PGKpuromycin (puro)* and *Gapd* as a loading control. Lanes were aligned electronically for better readability. ES cells were differentiated for 13 d in the presence of doxycycline (lane 1) or in the absence of inducer (lane 2). At the start of differentiation, parallel cultures received a *Xist* pulse for 24, 36, 48, or 60 h followed by withdrawal of inducer for the rest of the time (lanes 3 to 7) or followed by reinduction of *Xist* at day 8 of differentiation (lanes 8 to 11). All cells were analysed at day 13 of differentiation. (C) A quantitation of the *puro* expression relative to *Gapd* was derived from two independent Northern blots using tnimage software. A dashed red line indicates the 24-h interval in which the transition from reversible to irreversible silencing occurs.

We further determined at which time point in differentiation the chromosomal memory is established. For this, clone 36 ES cells were differentiated for 0, 12, 24, 36, 48, 60, or 72 h in the presence of doxycycline. Then *Xist* was turned off until day 8 of differentiation, when doxycycline was added back, and remethylation was assayed by immunofluorescence at day 13 in differentiation (Figure 7). In this experiment a transition occurred in a 24-h interval around 60 h if *Xist* was expressed for more than 48 h early in differentiation, allowing for efficient remethylation, a result consistent with the establishment of the memory in this interval. When *Xist* was turned off earlier than 60 h, remethylation was observed in only 10%–30% of the cells, demonstrating that the memory was not established. Turning off *Xist* at 72 h or later allowed remethylation in 85% of the cells. We also analysed the transition from *Xist*-dependent reversible to irreversible silencing in this experiment by Northern analysis of *puro* expression from the transgenic chromosome in differentiated clone 36 ES cells (Figure 7B). These data show that irreversible silencing was established in an interval between 48 and 72 h in ES cell differentiation, with *puro* expression levels dropping from 60% to 15% of the level in uninduced samples (Figure 7C), in agreement with our initial report (Wutz and Jaenisch 2000). The 24-h intervals for the transition can be explained by the asynchronous cell cycle states in the ES cell culture (doubling time, 21.4 h) at the time when differentiation was induced. We conclude that the establishment of the chromosomal memory is silencing independent and occurs at the time when X inactivation becomes irreversible and *Xist* independent.

## Discussion

Our results identify H3K27m3 and H4K20m1 as specific modifications that mark the *Xist*-expressing chromosome in undifferentiated ES cells and contribute to the epigenetic histone code of the Xi (Table 1). We did not observe an enrichment of H3K9m2 or H3K9m3 signals on the *Xist*-expressing chromosome, which has been reported by other studies. This could be a shortcoming of our transgenic system, but we also did not detect the H3K9m2 or H3K9m3 signals in female mouse primary embryonic fibroblasts (less than 2% of the cells). We attribute the different observations in other studies to the various antisera used. We supply peptide blot analysis for our antisera that suggest that the antibodies are highly specific (see Figure S1). This is also supported by the specific staining patterns in immunofluorescence experiments. The lysine 9 methylation signal observed in other studies could potentially be a result of cross reactivity with H3K27m3, a fact we can exclude for our H3K9 antibodies based on the staining pattern and peptide blots. Alternatively, our antibody might not recognise the H3K9m2 modification in the context of the chromosome. However, this is unlikely since the H3K9m3 signals for the pericentric regions and the Y chromosome are clearly identified. The H3K9m2 antiserum has been successfully used in ChIP analysis of the minor centromeric repeats (Yan et al. 2003) and reacts with these repeats in immunofluorescence, but does not show cross reactivity to H3K27m3. This suggests that our reagent is able to detect the modification in both ChIP and immunofluo-rescence experiments. Using highly specific antisera, we failed to see a strong signal for H3K9m2 in either ChIP or immunofluorescence experiments (see Figures 2 and S2E). In our ChIP analysis two chromosomal loci showed an increase for H3K9m2 upon *Xist* expression in differentiated ES cells, suggesting some enrichment for H3K9m2. We take these data to indicate that H3K9m2 is not a prominent mark of X inactivation but might be enriched locally to some degree upon differentiation.

Using *Xist* alleles that express a mutated version of *Xist,* which has a deletion of repeat A sequences and is unable to cause silencing, we showed that both H3K27m3 and H4K20m1 were established in the absence of transcriptional repression. This demonstrates that neither modification is sufficient to trigger silencing.

*Xist* expression led to rapid H3K27m3, which was complete after 1 to 2 d of *Xist* expression in both ES cells and differentiated cells (see Figure S3A and Figure 6, columns 1 and 2). This kinetics follows the localisation of *Xist* RNA, which accumulates between 4 and 12 h after doxycycline addition in ES cells (Wutz and Jaenisch 2000), suggesting that H3K27m3 is an immediate effect. We have further shown that in undifferentiated ES cells no progressive accumulation of the histone modifications occurs over time by comparing the percentage of cells showing H3K27m3, H4K20m1, and Ezh2 staining after 3 and 10 d expressing either full-length *Xist* RNA or a silencing-deficient mutant lacking repeat A (see Figure S3C). We have shown that H3K27m3 is a reversible modification throughout ES cell differentiation and depends at all stages on *Xist* expression. In undifferentiated ES cells H3K27m3 disappeared 48 h after *Xist* expression was turned off, corresponding to about two cell divisions. The kinetics would be consistent with the idea that replication is involved in the replacement of methylated histones, albeit our data do not rule out an active enzymatic process of demethylation. Importantly, we have observed nearly unchanged methylation levels 24 h after *Xist* expression has been turned off (see Figure S3B). This could reflect the intrinsic stability of the trimethylation mark or the persistence of the Eed/Ezh2 complex, which can stably associate with metaphase chromosomes from which *Xist* RNA is displaced (see Figure 1C; Mak et al. 2002). The transient maintenance of H3K27m3 might be significant for the mechanism of X inactivation. It could explain our observation that the inactive state will be “locked in” roughly 24 h after *Xist* loses its ability to initiate silencing, it will be locked in at 72 h of ES cell differentiation (Wutz and Jaenisch 2000).

Efficient methylation is established only when *Xist* expression is induced early in ES cell differentiation. The window in which *Xist* causes efficient methylation overlaps precisely with the initiation window, in which transcriptional silencing can be initiated. Yet methylation is independent of initiation of silencing. This would be consistent with the notion that H3K27m3 is necessary but not sufficient for silencing. However, this is unlikely, as a previous report has shown that in Eed mutant embryos, initiation of silencing is normal, but a defect in the maintenance of the inactive state leads to reactivation at later stages (Wang et al. 2001). Lower levels of Ezh2 and Eed could explain the restriction on the ability of *Xist* to induce H3K27m3 efficiently in differentiated ES cells (Silva et al. 2003). We do not favour this interpretation, as this restriction is observed at day 2 in differentiation, when Ezh2 and Eed protein levels are still high (see Figure 4C). Our data further show that the ability to efficiently methylate a chromosome late in ES cell differentiation is a feature of the chromosome and not a function of the protein levels of Eed and Ezh2. This is also in line with our observation that chromosome-wide H3K27m3 in clone 36 ES cells, in which Eed messenger RNA was reduced to 10%–15% of wild-type levels by stable RNAi, was still detected in 45%–60% of cells compared to 80% in control clone 36 cells (data not shown). Therefore, less abundant levels of Eed are sufficient to achieve efficient methylation. *Xist* induction later in ES cell differentiation or in cells of embryonic origin establishes H3K27m3 in only a small percentage of cells. The significance of H3K27m3 in this small number of cells is unclear at present.

**Figure 8:**
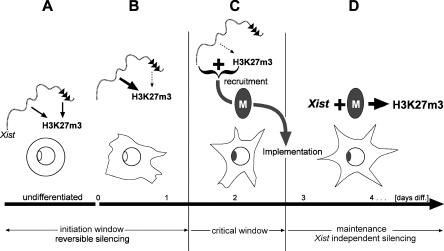
Model for the Transition from Initiation to Maintenance of X Inactivation Phases of X inactivation are given relative to days of ES cell differentiation (bottom). (A) In undifferentiated ES cells, efficient chromosome-wide H3K27m3 depends on both *Xist* RNA localisation to the chromosome in *cis* and initiation of transcriptional silencing via the A repeat (black triangles). (B) Early in differentiation, silencing becomes dispensable for high-level H3K27m3 (dotted arrow). (C) The beginning of the critical window is specified in that *Xist* loses its potential to trigger H3K27m3 (dotted arrow) and transcriptional silencing. The critical window is negotiated by sustaining high levels of H3K27m3, which is thought to constitute—together with *Xist* RNA—the signal for the recruitment of the chromosomal memory (black oval). The memory is established on the Xi exactly when silencing becomes irreversible and *Xist* independent. (D) During the maintenance phase of X inactivation the chromosomal memory allows *Xist* RNA to establish H3K27m3 efficiently.

The restriction of efficient methylation to early ES cell differentiation and the finding that methylation is reversible logically require that a chromosomal memory exists that enables H3K27m3 maintenance during differentiation. Previous models have suggested that a lock-in of X inactivation is based on chromosomal silencing, arguing that self-maintaining heterochromatin structures establish the principal form of memory. Our data clearly demonstrate that H3K27m3 is maintained in the absence of transcriptional repression, suggesting a chromosomal memory independent of silencing on the Xi. Using the inducible *Xist* expression system we have directly demonstrated the chromosomal memory (see Figure 6). A chromosome that had been exposed to *Xist* and been H3-K27 trimethylated early could be remethylated later in differentiation, after a period where *Xist* was turned off and methylation decayed, with significantly greater efficiency than a chromosome that had not expressed *Xist* early (see Figure 6). We have further determined the time point in ES cell differentiation when the chromosomal memory is established and found that it overlaps with the transition from *Xist*-dependent and reversible silencing to irreversible silencing. These data place the establishment of the memory in a critical phase of X inactivation. We note that the establishment of efficient H3K27m3 in the initiation window and the implementation of the memory are separated by a gap of approximately one cell division in ES cell differentiation. This parallels the gap between initiation of silencing and the maintenance of the silenced state independent of *Xist.* Our kinetic measurements indicate that H3K27m3 would decay from the *Xist*-expressing chromosome after two cell divisions; therefore, H3K27m3 could bridge the gap (critical window). We suggest that *Xist* expression and H3K27m3 might be the signal to recruit a chromosomal memory mediating the lock-in of X inactivation (Figure 8). In this model, silencing would be specified by separate signals depending on repeat A of *Xist,* which we predict would interact with the memory at the transition from reversible to irreversible and *Xist*-independent repression. In this regard we note that silencing or repeat A sequences enhance the efficiency of H3K27m3 in undifferentiated ES cells (see Figure 3B). However, there is no requirement for repeat A when ES cells are induced to differentiate (see Figures 6 and S3C). This could point to interactions between the silencing machinery and the Ezh2/Eed methylation complex specifically in ES cells.

The molecular basis for the chromosomal memory is presently unknown. Our data rule out the possibility that continuous *Xist* RNA expression or silencing is required for maintenance of the chromosomal memory and suggest that H3K27m3 is also not involved. The latter interpretation has to be treated cautiously, as it depends on the sensitivity of our assay to detect H3K27m3. Formally it is conceivable that low levels of H3K27m3 undetected by our assay could remain on the chromosome. Presently, it is also unclear what the role of H4K20m1 is and to what extent it interacts with H3K27m3. A H4-K20–specific histone methyltransferase has been identified (Fang et al. 2002; Nishioka et al. 2002; Rice et al. 2002), and we have performed in vitro functional analysis of the mouse Pr-Set7 protein (Figure S5; Protocol S1). Our results indicate that Pr-Set7 is a monomethylase for H4-K20. Its involvement in X inactivation and the function of H4K20m1 remain unclear at present. Future work is needed to identify the components of the memory configuration and to determine its precise function in X inactivation.

## Materials and Methods

### 

#### Cell lines, culture conditions, and histological sections.

Clone 36 ES cells (Wutz and Jaenisch 2000) and J1:XistΔSX-tetOP, T20:Xist, and ES cells expressing *Xist* deletions (Wutz et al. 2002) were cultured in DMEM (Biochrome, Berlin, Germany), 15% fetal calf serum (Euroclone, Milan, Italy), and 250 U of LIF/ml as described in those references. ES cells were induced to differentiate in ES medium without LIF by addition of all-*trans*-retinoic acid to 100 nM as described previously (Wutz and Jaenisch 2000). Primary mouse embryonic fibroblasts were derived from day 13.5 embryos and grown in DMEM (Biochrome) and 10% fetal calf serum as described previously (Wutz and Jaenisch 2000). *Xist* expression was induced by the addition of 1 μg/ml of doxycycline to the culture medium or was administered in drinking water (100 mg and 100 g of sucrose per liter). For sections, embryos were sexed (Lambert et al. 2000) and fixed, and 10-μm-thick frozen sections were prepared. Mice were handled according to institutional guidelines.

#### Immunostaining and Western blot.

For metaphase chromosome spreads, cells were incubated for 15 min at 37 °C in RBS solution (10 mM Tris-HCl [pH 7.5], 10 mM NaCl, 5 mM MgCl_2_), centrifuged for 10 min at 1,200 rpm onto Menzel SuperFrost slides (Roth, Karlsruhe, Germany) using a Cytospin 3 centrifuge (Thermo Shandon, Pittsburgh, Pennsylvania, United States). Staining was performed as described previously (Peters et al. 2003). Briefly, slides were extracted for 10 min at room temperature (RT) in KCM (10 mM Tris [pH 8.0], 120 mM KCl, 20 mM NaCl, 0.5 mM EDTA, 0.1% [vol/vol] Tween-20) containing 0.1% (vol/vol) Triton-X100, fixed for 10 min at RT in 2% PFA/PBS, washed in KCM/0.1% Tween-20, and blocked for 30 min at RT in KCM containing 2.5% (wt/vol) BSA, 0.1% Tween-20, and 10% normal goat serum (Jackson ImmunoResearch, West Grove, Pennsylvania, United States). Primary antibodies were diluted in blocking solution and incubated overnight at 4 °C. After washes in KCM/0.1% Tween-20, slides were incubated with secondary antibodies for 1 h at RT, washed, and mounted (Vectashield; Vector Laboratories, Burlingame, California, United States). For analysis of interphase nuclei, differentiated ES cells were grown on Roboz slides (CellPoint Scientific, Gaithersburg, Maryland, United States) and undifferentiated cells were attached to poly-l-lysine coated coverslips or cytospun as described above. Immunostaining was performed as described previously (Peters et al. 2003). Briefly, cells were fixed for 10 min at RT in 2% PFA in PBS, permeabilized for 5 min at RT in 0.1% Na Citrate/0.1% Triton-X100, blocked for 30 min at RT in PBS containing 2.5% (wt/vol) BSA, 0.1% Tween-20, and 10% normal goat serum, and processed as described above.

Antibodies for histone lysine methylation states are described elsewhere (Peters et al. 2003) and were used as follows (metaphase spreads/interphase): α-H3-K9m1 (IgG fraction of α-2x-monomethH3-K9, #4858, 1.7 mg/ml), 1:200/1:500; α-H3-K9m2 (IgG fraction of α-2x-dimeth H3-K9, #4679, 1.7 mg/ml), 1:100/1:200; α-H3-K9m3 (IgG fraction of α-2x-trimeth H3-K9, #4861, 1.3 mg/ml), 1:300/1:500; α-H3-K27m1 (IgG fraction of α-2x-monometh H3-K27, #8835, 0.7 mg/ml), 1:500/1:1,000; α-H3-K27m2 (IgG fraction of α-2x-dimeth H3-K27, #8841, 0.6 mg/ml), 1:500/1:1,000; α-H3-K27m3 (IgG fraction of α-2x-trimeth H3-K27, #6523, 1.1 mg/ml), 1:300/1:500. Additional antibodies were as follows: α-H3-K4m1 (α-monomethyl-Histone H3 [Lys4], #1799; Upstate Biotechnology, Lake Placid, New York, United States), 1:400/1:1,000; α-H3-K4m2 (α-dimethyl-Histone H3 [Lys4], #07-030; Upstate), 1:400/1:1,000; α-H3-K4m3 (α-trimethyl-Histone H3 [Lys4], #1819; Upstate), 1:700/1:1,000; α-H4-K20m1 (α-monomethyl-Histone H4 [Lys20], #07-440; Upstate), 1:100/1:200; α-H4-K20m2 (α-dimethyl-Histone H4 [Lys20], #07-367; Upstate), 1:200/1:200; α-H4-K20m3 (α-trimethyl-Histone H4 [Lys20], #07-463; Upstate), 1:350/1:500; polyclonal sheep α-H4Ac (Morrison and Jeppesen 2002), 1:500/1:1,000; polyclonal rabbit α-Ezh2 (Sewalt et al. 1998), 1:100/1:200. Secondary antibodies (Molecular Probes, Eugene, Oregon, United States) were as follows: Alexa A-11034 Fluor 488 goat antirabbit IgG (H+L), Alexa A-11036 Fluor 568 goat antirabbit IgG (H+L), and Alexa A-21099 Fluor 568 donkey antisheep IgG (H+L), all at 1:500.

For Western blots, total nuclear extract was separated by SDS PAGE, blotted onto a PVDF membrane (Immobilon-P; Millipore, Bedford, Massachusetts, United States), blocked in blocking solution (PBS, 3% [wt/vol] BSA), and incubated with primary antibodies for 3 h. After washing three times for 10 min in TBST (50 mM Tris-HCl [pH 8.0], 100 mM NaCl, 0.1% Tween 20) and incubation with secondary antibodies (HRP; Jackson Laboratory, Bar Harbor, Maine, United States), detection was performed using ECL reagent (Amersham Pharmacia Biotech, Little Chalfont, United Kingdom). Rabbit polyclonal α-Eed (1:3,500), rabbit polyclonal α-Ezh2 (1:1,000), goat polyclonal α-histone H3 (1:800, #sc-8654; Santa Cruz Biotechnology, Santa Cruz, California, United States), and rabbit polyclonal α-histone H4 (1:300, #07–108; Upstate) were used.

#### DNA FISH and RNA analysis.

For DNA FISH analysis, biotin-labelled STAR*FISH mouse whole chromosome-specific probes (1187-YMB-02, 1187–11MB-01; Cambio, Cambridge, United Kingdom) were detected with streptavidin, Alexa Fluor 633 conjugateS-21375 (Molecular Probes). RNA FISH probes were generated by random priming (Stratagene, La Jolla, California, United States) using Cy3-dCTP (Amersham). Hybridisation and washing were carried out as described previously (Wutz and Jaenisch 2000). Specimens were analysed using a fluorescence microscope (Zeiss Axioplan, Oberkochen, Germany) equipped with a CCD camera and the MetaMorph image analysis software (Universal Imaging, Downingtown, Pennsylvania, United States). Northern analysis was performed using 20 μg of RNA (Trizol; Invitrogen, Carlsbad, California, United States) as described previously (Wutz et al. 2002).

#### ChIPs.

Cells were cross-linked with 1% formaldehyde for 10 min at RT and quenched with 125 mM glycine, and whole-cell extracts were prepared. ChIPs were performed in duplicates as described previously (Martens et al. 2003). Briefly, 400 μg of fragmented chromatin (between 400 and 1000 base pairs) was used for immunoprecipitation, and DNA was extracted from the precipitates and analysed by real-time PCR using a Lightcycler (Roche Diagnostics, Basel, Switzerland). Results were corrected for nonspecific binding to the beads and presented as a percentage of the input DNA (4 μg of fragmented chromatin, 100%). Primers sequences were as follows: tubulin, CCTGCTGGGAGCTCTACT and GGGTTCCAGGTCTACGAA; puromycin, GCTGCAAGAACTCTTCCTC and GCCTTCCATCTGTTGCTG; d11mit117, AAAAGACCCTATTTACAATACAACTGA and TGTCATTTTTGATTAATCGCTCC; d11mit108, GGCACAAGAAAGACACAGCA and AAAGAGAAACCCCAGAGGGA; d11mit102, CCAGGAGAGCAGGAAGGTC and TCCTTCTGGGTGCTGCAT; d15mit15, AGCATACACTCTTGTTCCTGCT and AATAAATACCAGAGAAGCACCGTG.

## Supporting Information

Figure S1 Specificities of H3-K9, H3-K27, H4-K20, and H3-K4 Mono-, Di-, and Trimethyl Antibodies Immunodotblot analysis (Peters et al. 2003) of the antisera used to detect specific methylation states of histone H3 on Lysine 9 (A), H3 on Lysine 27 (B), H4 on Lysine 20 (C), and H3 on Lysine 4 (D). IgG fractions of the methyl-lysine histone antibodies were tested at various dilutions, with the most optimal dilution being displayed. Dotblots contain 0.4, 2, 10, and 50 pmol of linear H3 (amino acids 1–20; amino acids 19–34; amino acids 25–45; amino acids 72–91) and peptides, either unmodified or mono-, di-, or trimethylated at the K4, K9, K27, K36, or K79 positions. In addition, a linear H4 (amino acids 12–31) peptide, mono-, di-, or trimethylated at the K20 position, was also used.
(611 KB PDF).

Figure S2 Histone Modification Pattern of the Inactive X Chromosome Immunofluorescence staining of metaphase spreads of clone 36 ES cells induced to express *Xist* for 3 d using H3K27m1 (A), H3K27m2 (B), H3K27m3 (C), H3K9m1 (D), H3K9m2 (E), H3K9m3 (F), H4K20m1 (G), H4K20m2 (H), H4K20m3 (I), H3K4m3 (J), and H4Ac (K) antisera. Chromosome 11 was identified by a DNA FISH probe (red; blue, DAPI) in (J) and (K). Clone 36 ES cells grown in the absence of doxycycline are used as a control for the H4Ac staining without *Xist* expression (L).
(4.8 MB TIF).

Figure S3 Initiation and Maintenance of Histone Methylation during Differentiation (A) The kinetics of H3K27m3 was measured in undifferentiated clone 36 ES cells. The number of cells showing H3K27m3 staining 6, 12, 24, and 48 h after induction of *Xist* expression is shown.
(B) The stability of H3K27m3 was determined in undifferentiated ES cells. The percentage of metaphase chromosome spreads (*n* > 150) showing H3K27m3 staining was analysed in undifferentiated clone 36 ES cells, which expressed *Xist* for 3 d (lane 1) or were further grown without inducer for 24 h (lane 2) or 48 h (lane 3). This experiment complements data presented in Figure 5A and 5B providing a ‘cell cycle synchronous' view of the H3K27m3 decay kinetics.
(C) Levels of H3K27m3 were measured in undifferentiated ES cells after 3 and 10 d of *Xist* expression. No progressive accumulation over time was observed, indicating that the steady state of H3K27m3 has been reached at 3 d *Xist* expression. However, a marked increase in methylation is observed in J1:XistΔSX-tetOP ES cells upon differentiation for 2 d (hatched bar).
(D) Combined Xist RNA FISH (red) immunofluorescence analysis of Ezh2 and H4K20m1 in undifferentiated J1:XistΔSX-tetOP cells expressing *Xist* for 3 and 10 d (percentage of nuclei showing a staining is given). Analysis of H3K27m3 and H4 acetylation using an antiserum specific for multiply acetylated forms of H4 in clone 36 and J1:XistΔSX-tetOP ES cells that were grown for 4 d in the presence of doxycycline and then shifted to differentiation conditions for 2 d more in the presence of doxycylcine.
(E) Male primary mouse fibroblasts (PMEFs) hemizygous for the inducible Xist-tetOP allele and homozygous for the tetracycline-inducible transactivator were induced with doxycycline for 2 d (lane 1) or 3 d (lane 2), and the number of cells showing H3K27m3 staining in interphase was analysed. Control female PMEFs showed a methylation signal in the large majority of cells (lane 3); uninduced male PMEFs were always negative.
(F) Representative indirect immunofluorescence of uninduced (top) and induced (bottom) male Xist-tetOP PMEFs. The inducible *Xist* RNA triggers less pronounced and less dense foci of H3-K27 trimethylation (green) compared to the female wild-type control.
(G) Upon *Xist* expression, H4-K20 monomethylation (green) is observed in interphase Xist-tetOP PMEFs (left). Focal enrichment colocalises with the site or *Xist* RNA clusters (red) on the X chromosome. Female wild-type PMEFs (right).
(3.0 MF TIF).

Figure S4 Analysis of the XistΔXSa Mutation The XistΔXSa transgene was integrated by Cre-mediated recombination into the Hprt locus on the single X chromosome in T20 ES cells (Wutz et al. 2002). A schematic representation of the *Xist* cDNA in given (top): repeats A to E are indicated by arrays of triangles, sequences mediating localisation to chromatin are indicated by boxes underneath (degree of hatching represents importance), and the location of the deletion is indicated by a coloured box. RNA localisation was analysed by FISH (lower left), showing that the RNA localises in small clusters in some cells. The ability of the RNA to induce silencing was measured by cell survival of differentiating cultures under induced versus uninduced conditions (lower right). Controls are cells either having a fully functional *Xist* cDNA transgene *(Xist)* or a cDNA lacking repeat A that is incompetent to induce silencing *(ΔSX).* The *ΔXSa* RNA shows poor silencing activity, presumably as a consequence of its failure to localise well to the chromosome.
(1.5 MB TIF).

Figure S5 Selective H4-K20 Monomethylation Activity of Mouse Pr-Set7 In Vitro (A) Schematic presentation of full-length mouse *PR/SET domain-containing protein 07 (Pr-Set7),* indicating SET domain in black (gi:38080595). Below, region tested for histone methyltransferase (HMTase) activity.
(B) Coomassie stain (left) shows purified recombinant GST-tagged *Pr-Set7* (arrow), H4 peptides (arrowhead), and histones used for in vitro reactions with S-adenosyl-[methyl-^14^C]-L-methionine as methyl donor. Fluorography (right) indicates HMTase activity on the unmodified H4 peptide comprising residues 12–31 of the histone H4 N-terminus. Notably, no further methyl groups could be transferred to the same peptide if it had been synthetically monomethylated at residue H4 lysine 20 (K20m1) before usage in the in vitro reaction. Free histones are not accepted as substrate.
(C) Fluorography indicates histone H4 HMTase activity of *GST–Pr-Set7* selective for the unmodified histone H4 peptide (12–31). H4-K20 monomethylation obviously is the terminal state for *Pr-Set7,* because synthetically mono- (K20m1), di- (K20m2), and trimethylated H4 peptides (K20m3) could not be significantly methylated.
(948 KB TIF).

Protocol S1 Supplementary Methods (22 KB DOC).

## Abbreviations

ChIP: chromatin immunoprecipitation
ES cell: embryonic stem cell
FISH: fluorescent in situ hybridization
H3-K9: histone H3 lysine 9
H3K27m3: histone H3 lysine 27 trimethylation
HMTase: histone methyltransferase
PMEF: primary mouse fibroblast
RT: room temperature
Xi: inactive X chromosome
