# Supplementary material for: A Chromosomal Memory Triggered by Xist Regulates Histone Methylation in X Inactivation
Source: PLoS Biol. 2004 Jul 13;2(7):e171. doi: 10.1371/journal.pbio.0020171 (PMC449785; doi:10.1371/journal.pbio.0020171)
Supplement: Figure S1 — Immunodotblot analysis (Peters et al. 2003) of the antisera used to detect specific methylation states of histone H3 on Lysine 9 (A), H3 on Lysine 27 (B), H4 on Lysine 20 (C), and H3 on Lysine 4 (D). IgG fractions of the methyl-lysine histone antibodies were tested at various dilutions, with the most optimal dilution being displayed. Dotblots contain 0.4, 2, 10, and 50 pmol of linear H3 (amino acids 1–20; amino acids 19–34; amino acids 25–45; amino acids 72–91) and peptides, either unmodified or mono-, di-, or trimethylated at the K4, K9, K27, K36, or K79 positions. In addition, a linear H4 (amino acids 12–31) peptide, mono-, di-, or trimethylated at the K20 position, was also used. (611 KB PDF). [file pbio.0020171.sg001.pdf]

H3-K9 methylation antibodies

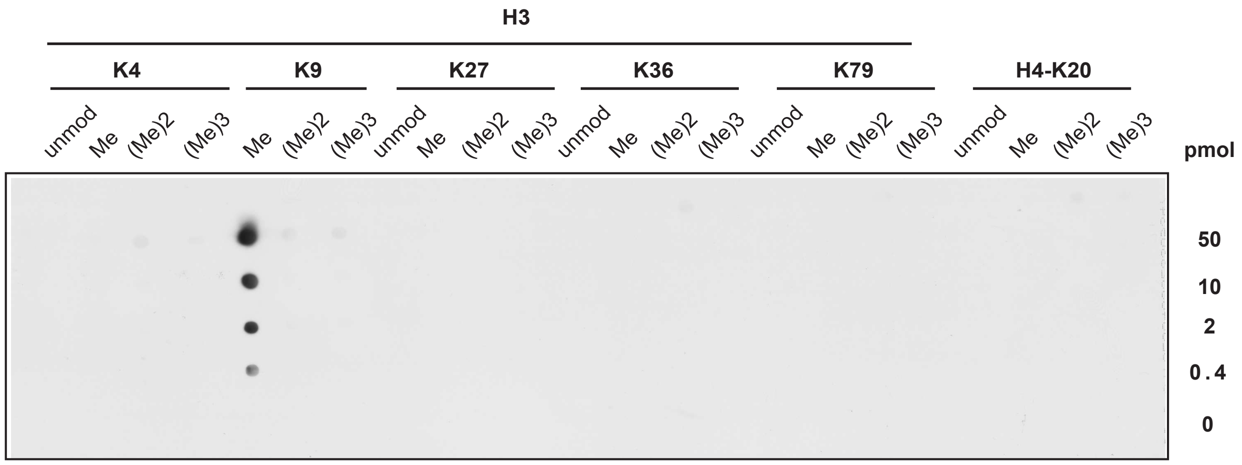

α-2x-mono-meth H3-K9
#4858, IgG, 1:2500

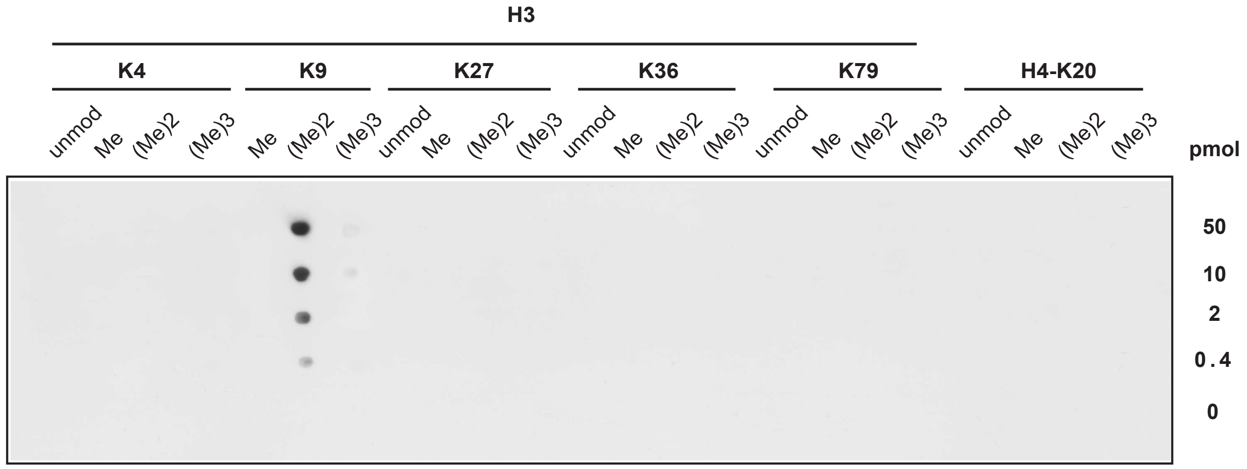

α-2x-di-meth H3-K9
#4679, IgG, 1:2500

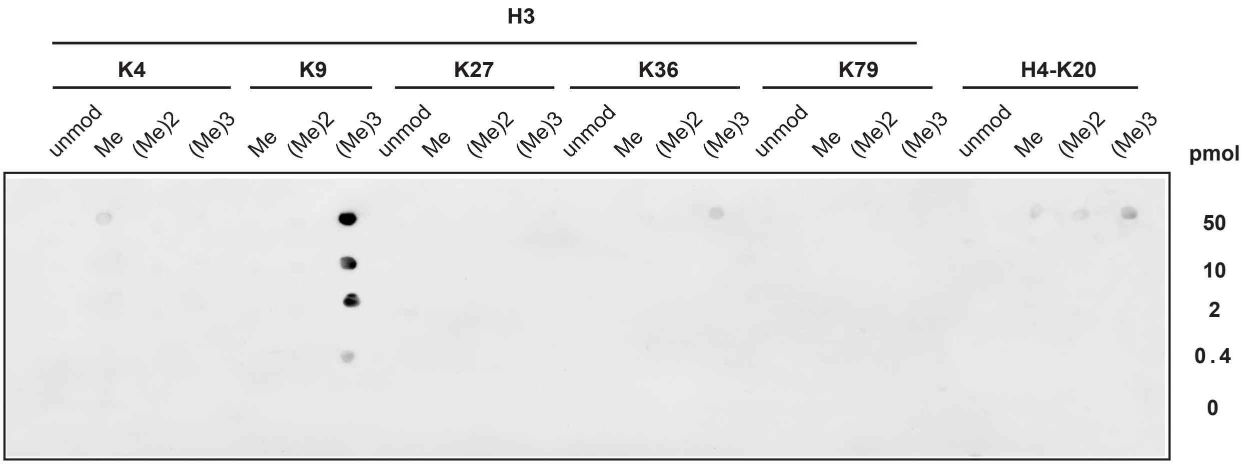

α-2x-tri-meth H3-K9
#4861, IgG, 1:2500

Kohlmaier et al.
Suppl. 1A

Peters et al., 2003

H3-K27 methylation antibodies

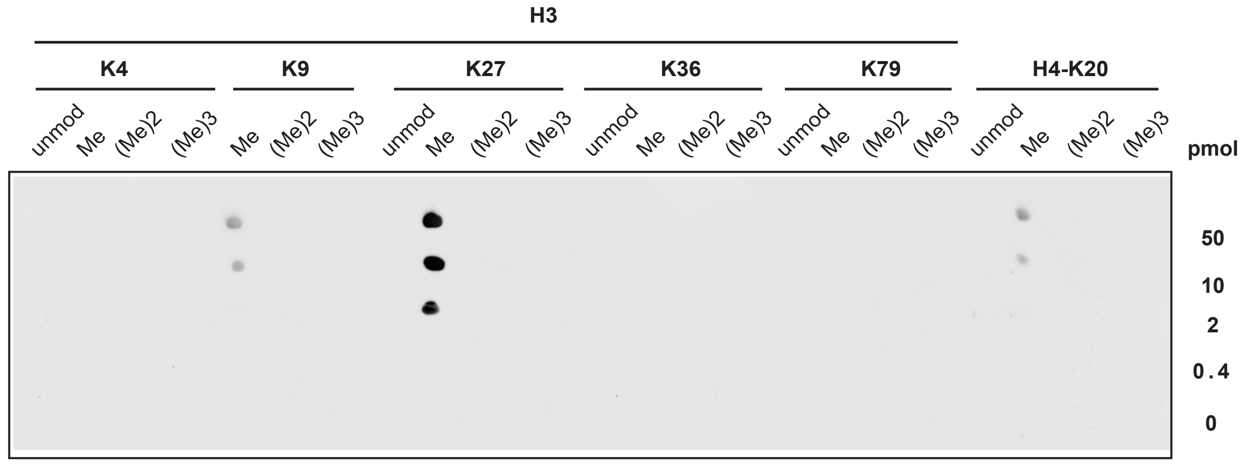

α-2x-mono-meth H3-K27
#8835, IgG, 1:2500

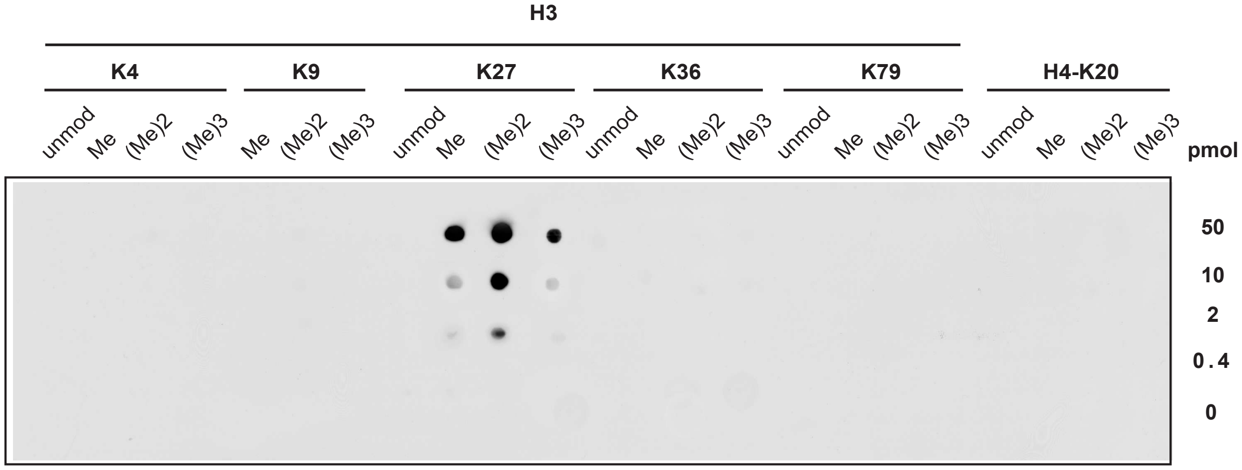

α-2x-di-meth H3-K27
#8841, IgG, 1:1000

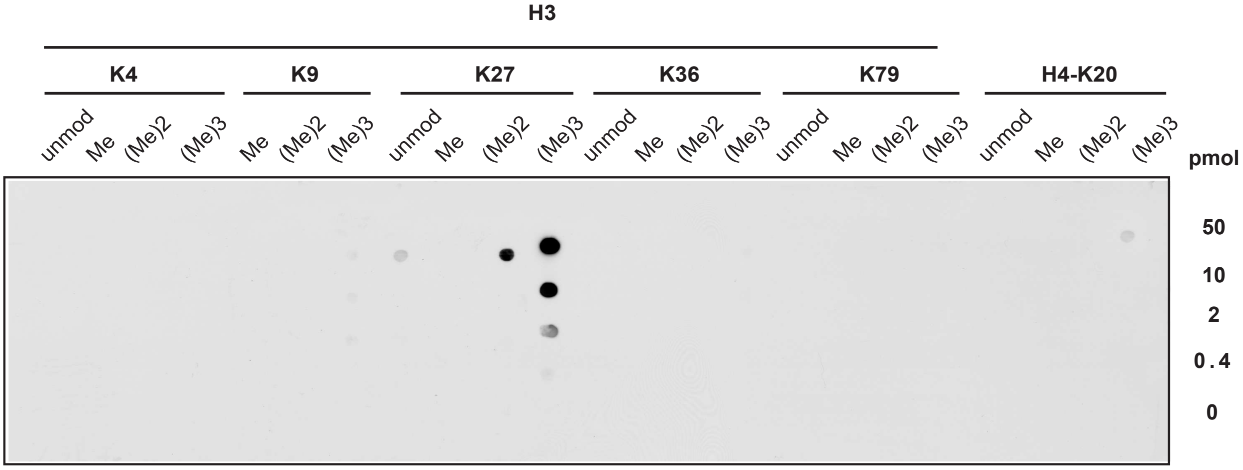

α-2x-tri-meth H3-K27
#6523, IgG, 1:1500

Kohlmaier et al.
Suppl. 1B

Peters et al., 2003

H4-K20 methylation antibodies

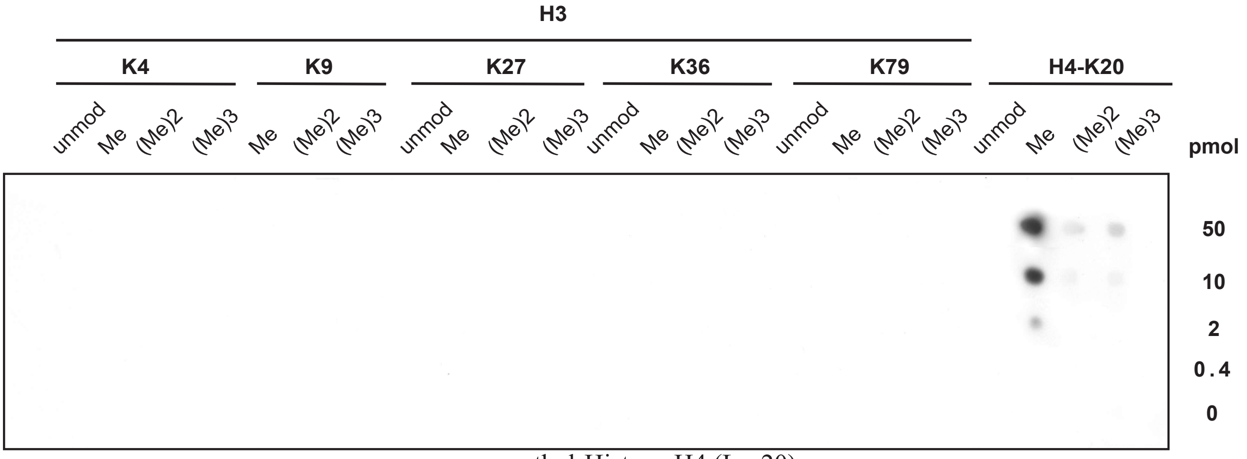

α-monomethyl-Histone H4 (Lys20)
07-440, 1:1000

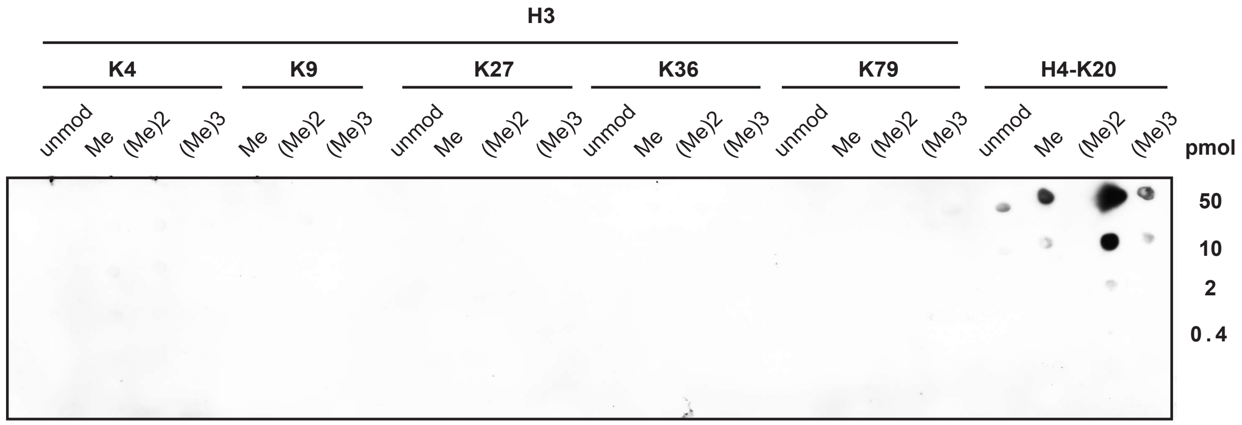

α-dimethyl-Histone H4 (Lys20)
07-367, 1:1000

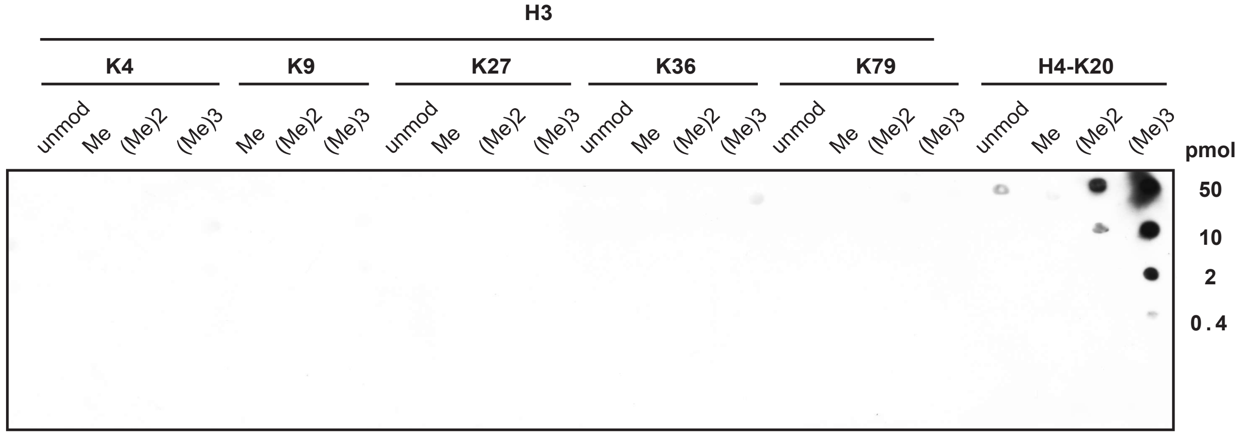

α-trimethyl-Histone H4 (Lys20)
#07-463, 1:1000

Kohlmaier et al.
Suppl. 1C

UBI

H3-K4 methylation antibodies

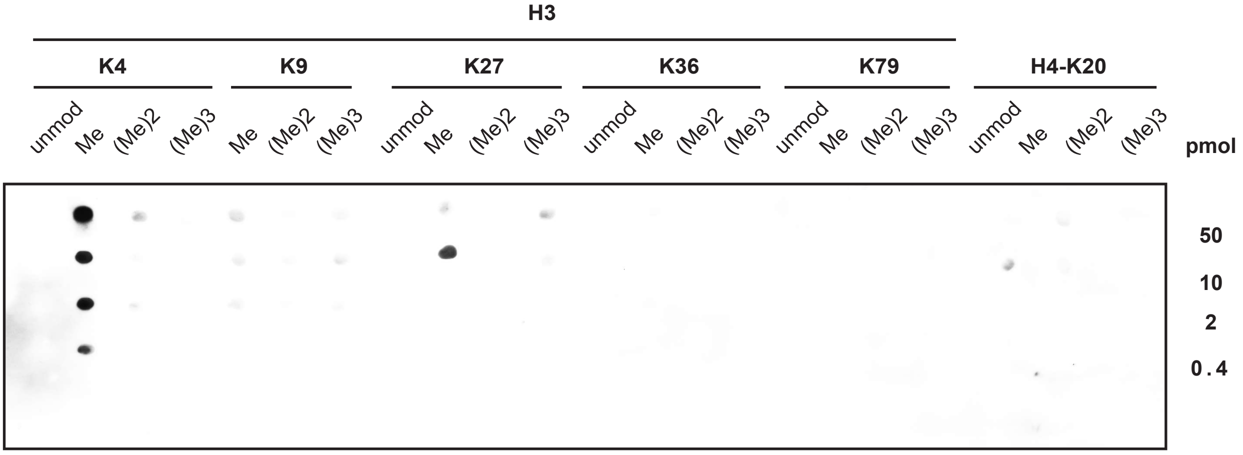

α-monomethyl-Histone H3 (Lys4)
1799, 1:1000

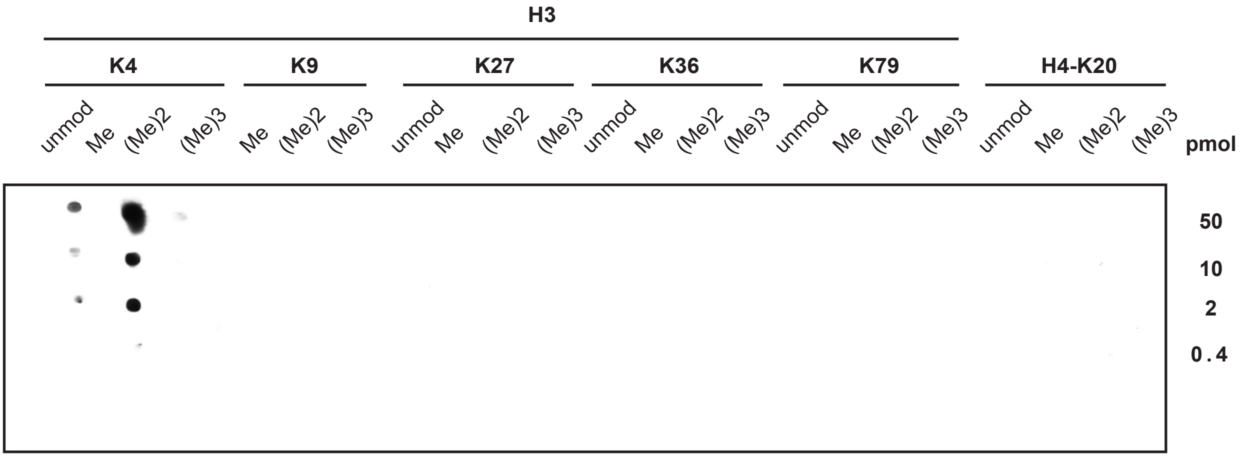

α-dimethyl-Histone H3 (Lys4)
07-030, 1:1000

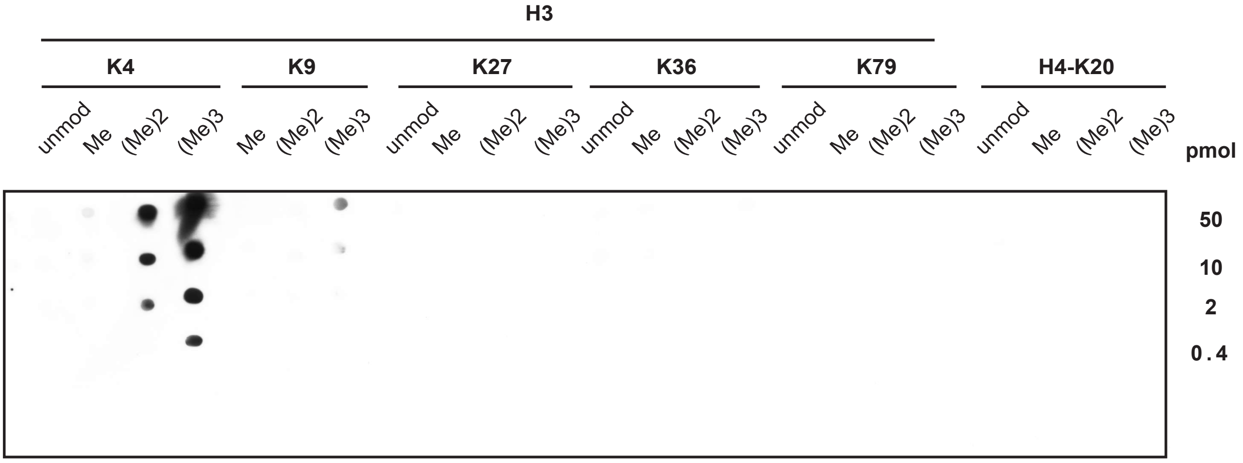

α-trimethyl-Histone H3 (Lys4)
#1819, 1:1000

Kohlmaier et al.
Suppl. 1D

UBI
