# Supplementary figures and images for: A Chromosomal Memory Triggered by Xist Regulates Histone Methylation in X Inactivation
Source: PLoS Biol. 2004 Jul 13;2(7):e171. doi: 10.1371/journal.pbio.0020171 (PMC449785; doi:10.1371/journal.pbio.0020171)

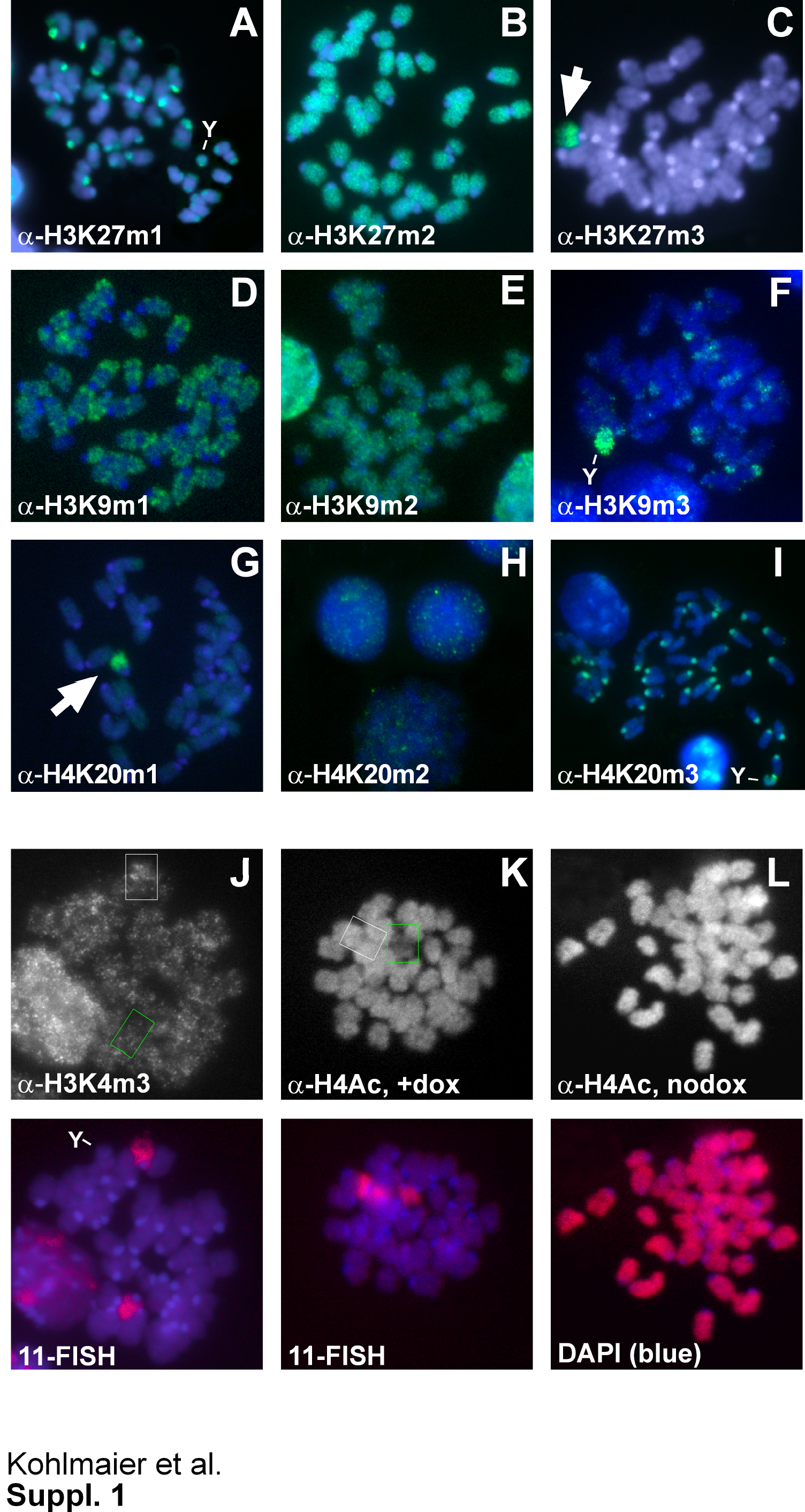

Supplement: Figure S2 — Immunofluorescence staining of metaphase spreads of clone 36 ES cells induced to express Xist for 3 d using H3K27m1 (A), H3K27m2 (B), H3K27m3 (C), H3K9m1 (D), H3K9m2 (E), H3K9m3 (F), H4K20m1 (G), H4K20m2 (H), H4K20m3 (I), H3K4m3 (J), and H4Ac (K) antisera. Chromosome 11 was identified by a DNA FISH probe (red; blue, DAPI) in (J) and (K). Clone 36 ES cells grown in the absence of doxycycline are used as a control for the H4Ac staining without Xist expression (L). (4.8 MB TIF). [file pbio.0020171.sg002.tif]

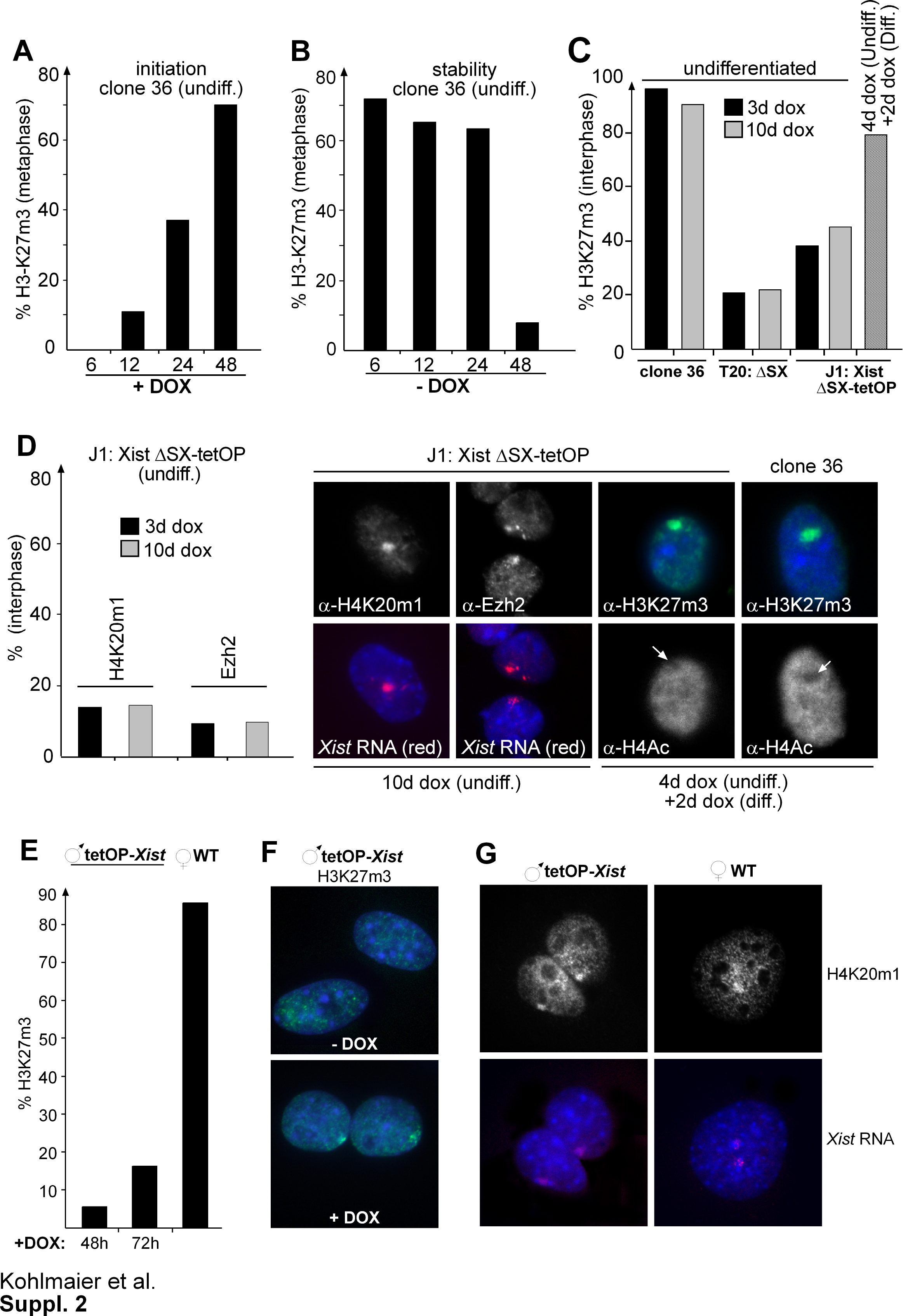

Supplement: Figure S3 — (A) The kinetics of H3K27m3 was measured in undifferentiated clone 36 ES cells. The number of cells showing H3K27m3 staining 6, 12, 24, and 48 h after induction of Xist expression is shown. (B) The stability of H3K27m3 was determined in undifferentiated ES cells. The percentage of metaphase chromosome spreads (n > 150) showing H3K27m3 staining was analysed in undifferentiated clone 36 ES cells, which expressed Xist for 3 d (lane 1) or were further grown without inducer for 24 h (lane 2) or 48 h (lane 3). This experiment complements data presented in Figure 5A and 5B providing a ‘cell cycle synchronous' view of the H3K27m3 decay kinetics. (C) Levels of H3K27m3 were measured in undifferentiated ES cells after 3 and 10 d of Xist expression. No progressive accumulation over time was observed, indicating that the steady state of H3K27m3 has been reached at 3 d Xist expression. However, a marked increase in methylation is observed in J1:XistΔSX-tetOP ES cells upon differentiation for 2 d (hatched bar). (D) Combined Xist RNA FISH (red) immunofluorescence analysis of Ezh2 and H4K20m1 in undifferentiated J1:XistΔSX-tetOP cells expressing Xist for 3 and 10 d (percentage of nuclei showing a staining is given). Analysis of H3K27m3 and H4 acetylation using an antiserum specific for multiply acetylated forms of H4 in clone 36 and J1:XistΔSX-tetOP ES cells that were grown for 4 d in the presence of doxycycline and then shifted to differentiation conditions for 2 d more in the presence of doxycylcine. (E) Male primary mouse fibroblasts (PMEFs) hemizygous for the inducible Xist-tetOP allele and homozygous for the tetracycline-inducible transactivator were induced with doxycycline for 2 d (lane 1) or 3 d (lane 2), and the number of cells showing H3K27m3 staining in interphase was analysed. Control female PMEFs showed a methylation signal in the large majority of cells (lane 3); uninduced male PMEFs were always negative. (F) Representative indirect immunofluorescence of uninduced (top [file pbio.0020171.sg003.tif]

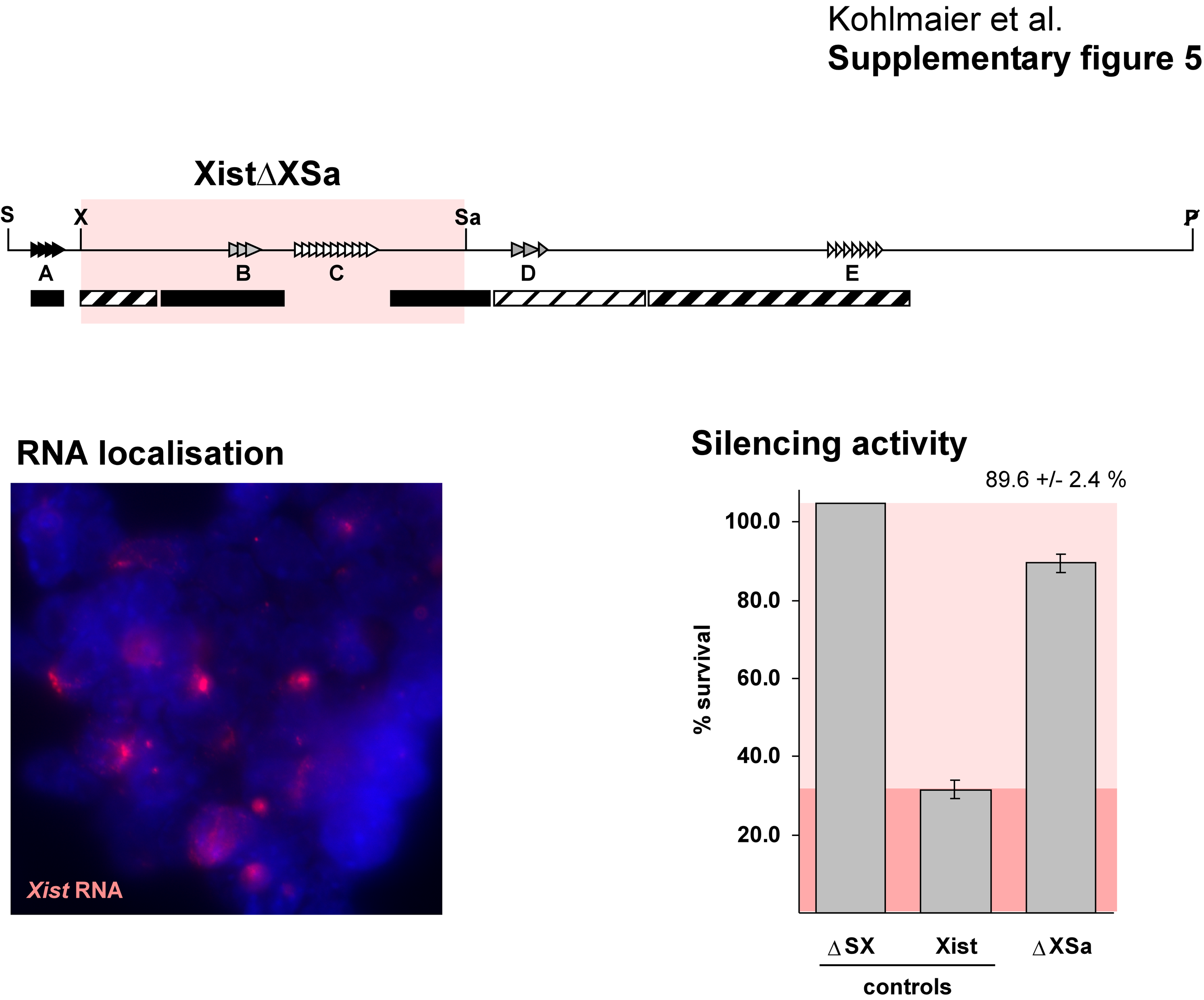

Supplement: Figure S4 — The XistΔXSa transgene was integrated by Cre-mediated recombination into the Hprt locus on the single X chromosome in T20 ES cells (Wutz et al. 2002). A schematic representation of the Xist cDNA in given (top): repeats A to E are indicated by arrays of triangles, sequences mediating localisation to chromatin are indicated by boxes underneath (degree of hatching represents importance), and the location of the deletion is indicated by a coloured box. RNA localisation was analysed by FISH (lower left), showing that the RNA localises in small clusters in some cells. The ability of the RNA to induce silencing was measured by cell survival of differentiating cultures under induced versus uninduced conditions (lower right). Controls are cells either having a fully functional Xist cDNA transgene (Xist) or a cDNA lacking repeat A that is incompetent to induce silencing (ΔSX). The ΔXSa RNA shows poor silencing activity, presumably as a consequence of its failure to localise well to the chromosome. (1.5 MB TIF). [file pbio.0020171.sg004.tif]

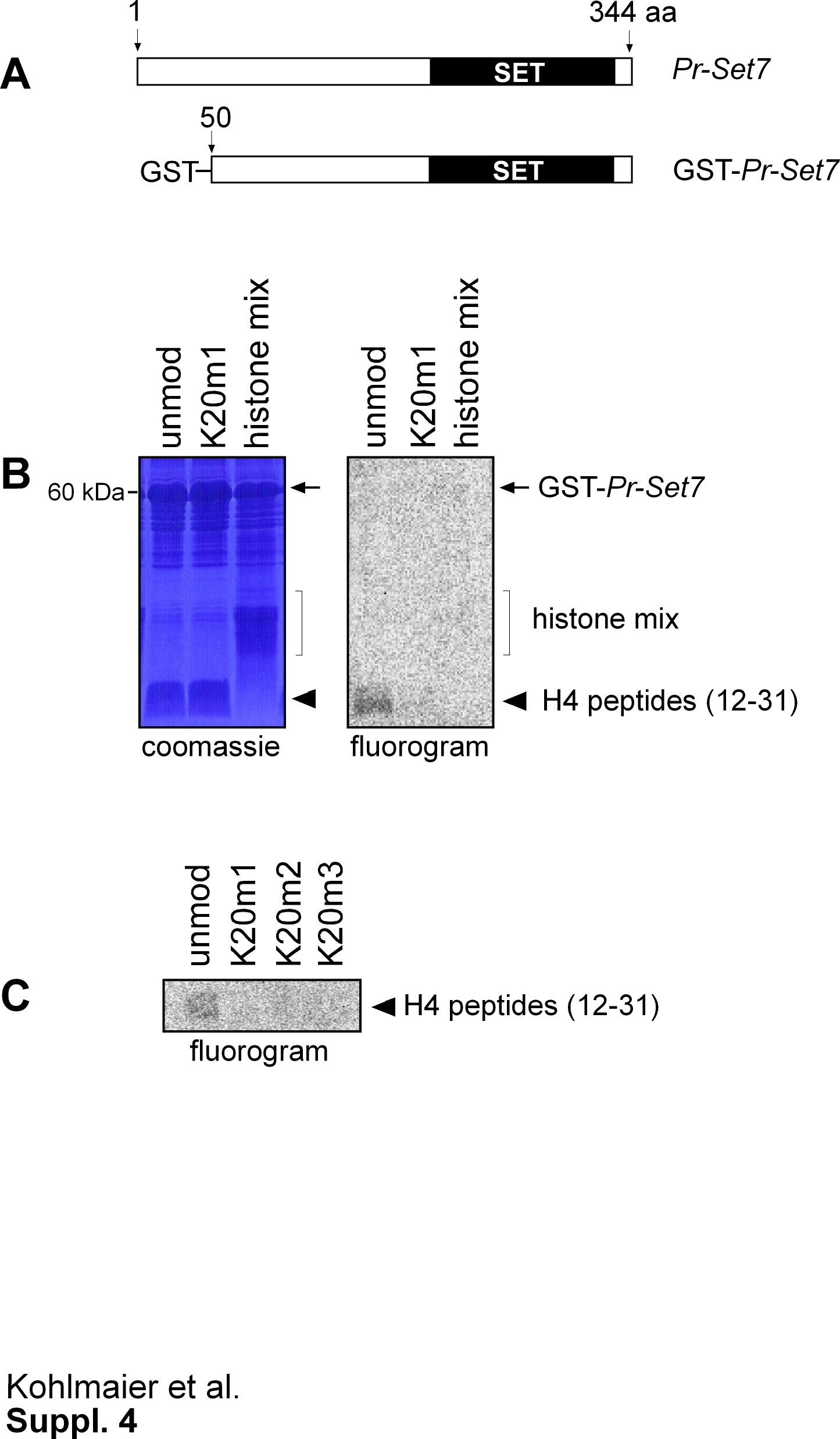

Supplement: Figure S5 — (A) Schematic presentation of full-length mouse PR/SET domain-containing protein 07 (Pr-Set7), indicating SET domain in black (gi:38080595). Below, region tested for histone methyltransferase (HMTase) activity. (B) Coomassie stain (left) shows purified recombinant GST-tagged Pr-Set7 (arrow), H4 peptides (arrowhead), and histones used for in vitro reactions with S-adenosyl-[methyl-14C]-L-methionine as methyl donor. Fluorography (right) indicates HMTase activity on the unmodified H4 peptide comprising residues 12–31 of the histone H4 N-terminus. Notably, no further methyl groups could be transferred to the same peptide if it had been synthetically monomethylated at residue H4 lysine 20 (K20m1) before usage in the in vitro reaction. Free histones are not accepted as substrate. (C) Fluorography indicates histone H4 HMTase activity of GST–Pr-Set7 selective for the unmodified histone H4 peptide (12–31). H4-K20 monomethylation obviously is the terminal state for Pr-Set7, because synthetically mono- (K20m1), di- (K20m2), and trimethylated H4 peptides (K20m3) could not be significantly methylated. (948 KB TIF). [file pbio.0020171.sg005.tif]
