# Supplementary material for: A Chromosomal Memory Triggered by Xist Regulates Histone Methylation in X Inactivation
Source: PLoS Biol. 2004 Jul 13;2(7):e171. doi: 10.1371/journal.pbio.0020171 (PMC449785; doi:10.1371/journal.pbio.0020171)
Supplement: Protocol S1 — (22 KB DOC). [file pbio.0020171.sd001.doc]

**Supplementary Methods**

# Generation and purification of GST fusion protein

Pr-Set7 was PCR amplified from IMAGE Clone IMAGp998P2111880 (RZPD Deutsches Ressourcenzentrum für Genomforschung), expressed as N-terminal GST fusion protein from Gateway vector pDEST 15 (Invitrogen) and purified as described before (Rea et al., 2000; Nature 406:593-599).

# *In vitro* histone methyltransferase assay

In vitro HMTase reactions were performed in 55μl of methylase activity buffer (50mM glycine pH 9,3, 250mM sucrose and 2mM DTT) containing 20 μg of synthetic linear peptides or of free histones (mixture of H1, H3, H2B, H2A and H4; Boehringer Mannheim) as substrate and 50 nCi S-adenosyl-[methyl-^14^C]-L-methionine (Amersham) as methyl donor. Approximately 50 µg of matrix bound GST-fusion protein was used during an overnight incubation at 37°C. Reactions were stopped by boiling in SDS loading buffer, peptides were resolved by SDS-PAGE and visualized by Coomassie staining and subsequent fluorography. Peptides used: histone H4 (12-31, unmodified) KGGAKRHRKVLRDNIQGITK-cys, histone H4 (12-31, mono-methylated at K20) KGGAKRHRK(Me)VLRDNIQGITK-cys, histone H4 (12-31, di-methylated at K20) KGGAKRHRK(Me2)VLRDNIQGITK-cys, histone H4 (12-31, tri-methylated at K20) KGGAKRHRK(Me3)VLRDNIQGITK-cys.

**Immuno-dotblots**

Increasing amounts (0.4, 2, 10 and 50 pmoles dissolved in 2 μl PBS) of different peptides as indicated in supplementary figures were spotted onto PVDF membranes (Hybond-P, Amersham Bioscience) and dried o/n at RT. Membranes were subsequently blocked in blocking solution (PBS, 3% (w/v) BSA) for 1 hour, incubated with primary antibodies for 1 hour and washed 5 times for 10 min in TBST (50 mM Tris-HCl

pH 8.0, 100 mM NaCl, 0.1% (v/v) Tween 20). Blots were exposed to secondary antibodies (HRP; Jackson Laboratories) for 45 min in PBS containing 5% (w/v) non-fat dry milk powder. After 5 washes for 10 min in TBST the blots were developed using a two component ECL detection reagent (Amersham Bioscience) and exposed to scientific imaging film (Kodak X-Omat Blue XB-1).
